# Rechargeable aqueous zinc-manganese dioxide batteries with high energy and power densities

**DOI:** 10.1038/s41467-017-00467-x

**Published:** 2017-09-01

**Authors:** Ning Zhang, Fangyi Cheng, Junxiang Liu, Liubin Wang, Xinghui Long, Xiaosong Liu, Fujun Li, Jun Chen

**Affiliations:** 1 Key Laboratory of Advanced Energy Materials Chemistry (Ministry of Education) and State Key Laboratory of Elemento-Organic Chemistry, College of Chemistry, Nankai University, Tianjin, 300071 China; 2 Collaborative Innovation Center of Chemical Science and Engineering, Nankai University, Tianjin, 300071 China; 3 State Key Laboratory of Functional Materials for Informatics, Shanghai Institute of Microsystem and Information Technology, Chinese Academy of Sciences, Shanghai, 200050 China

## Abstract

Although alkaline zinc-manganese dioxide batteries have dominated the primary battery applications, it is challenging to make them rechargeable. Here we report a high-performance rechargeable zinc-manganese dioxide system with an aqueous mild-acidic zinc triflate electrolyte. We demonstrate that the tunnel structured manganese dioxide polymorphs undergo a phase transition to layered zinc-buserite on first discharging, thus allowing subsequent intercalation of zinc cations in the latter structure. Based on this electrode mechanism, we formulate an aqueous zinc/manganese triflate electrolyte that enables the formation of a protective porous manganese oxide layer. The cathode exhibits a high reversible capacity of 225 mAh g^−1^ and long-term cyclability with 94% capacity retention over 2000 cycles. Remarkably, the pouch zinc-manganese dioxide battery delivers a total energy density of 75.2 Wh kg^−1^. As a result of the superior battery performance, the high safety of aqueous electrolyte, the facile cell assembly and the cost benefit of the source materials, this zinc-manganese dioxide system is believed to be promising for large-scale energy storage applications.

## Abstract (web summary)

The development of rechargeable aqueous zinc batteries are challenging but promising for energy storage applications. With a mild-acidic triflate electrolyte, here the authors show a high-performance Zn-MnO_2_ battery in which the MnO_2_ cathode undergoes Zn^2+^ (de)intercalation.

## Introduction

There is ever increasing demand of advanced battery technologies with high safety and low cost for applications in portable electronics, electrified vehicles, and renewable energy storage^1–7^. Although lithium-ion batteries have gained great improvement in energy/power density and life span, the safety issues associated with flammable organic electrolytes and the growing concerns of the price and availability of Li resources impede their large-scale deployment. Battery chemistries based on electrochemical intercalation/storage of Na^+^, K^+^, Mg^2+^, and Zn^2+^ in aqueous electrolytes have been considered as promising alternatives, because of high safety, materials abundance, and environmental friendliness^8–19^. Rechargeable Zn-ion batteries (ZIBs) are particularly attractive as zinc features higher water compatibility and stability than alkaline metals, allows multivalent charge transport carriers, and can be produced and recycled with mature industrial process^20–25^.

**Fig. 1:**
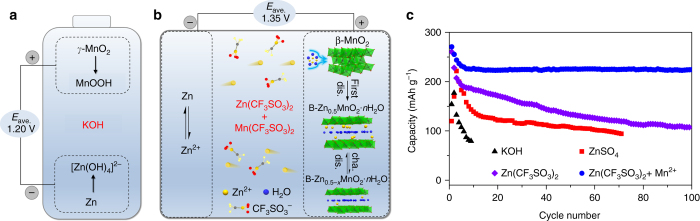
Zn-MnO_2_ battery chemistry. Schematic illustration of **a** the primary alkaline Zn-MnO_2_ battery using KOH electrolyte and **b** the rechargeable Zn-MnO_2_ cell using CF_3_SO_3_^−^-based electrolyte. **c** Comparison of the cycling performance of Zn-MnO_2_ cells with electrolytes of 45wt.% KOH (at 0.32 C), 3 M ZnSO_4_, 3 M Zn(CF_3_SO_3_)_2_, and 3 M Zn(CF_3_SO_3_)_2_ with 0.1 M Mn(CF_3_SO_3_)_2_ additive at 0.65 C. *n*C equals the rate to charge/discharge the thereotical capacity (308 mAh g^−1^) of MnO_2_ in 1/*n* hours

Zinc-manganese dioxide (Zn-MnO_2_) batteries have dominated the primary battery market because of low cost, high safety, and easy manufacturing^26–28^. It is highly intriguing to develop rechargeable Zn-MnO_2_ batteries. Nevertheless, previous attempts are plagued by poor cycling performance due to the formation of irreversible discharged species (e.g., Mn(OH)_2_ and ZnO at cathode and anode, respectively) in alkaline electrolytes^29–31^. Although alkaline Zn-MnO_2_ batteries (Fig. 1a) were shown rechargeable for extended cycles, the delivered capacity is limited at shallow depth of discharge (~ 10%)^32^. Recently, the rechargeability of aqueous Zn-MnO_2_ batteries has been improved by using mild acidic electrolyte (e.g., aqueous ZnSO_4_ solution)^33−37^. However, the reaction mechanism of MnO_2_ polymorphs remains elusive and controversial. For example, electrochemical Zn-insertion in α-MnO_2_ is shown to undergo phase transition from tunneled structure to spinel ZnMn_2_O_4_^33^, layered Zn-buserite^36^, or birnessite^38^, most of which collapse upon cycling. A different mechanism was referred to the conversion reaction between α-MnO_2_ and MnOOH^35^. For γ-MnO_2_, complex mutiple-phase transformation was proposed on discharge, involving spinel-type ZnMn_2_O_4_, tunnel-type γ-Zn_*x*_MnO_2_, and layered-type L-Zn_*x*_MnO_2_^34^. Additionally, the Zn-insertion properties in aqueous ZnSO_4_ electrolyte are found to vary among polymorphs: α-MnO_2_ featuring (2 × 2) + (1 × 1) tunnel structure^39^ and γ-MnO_2_ with (1 × 2) + (1 × 1) tunnels exhibit high capacity (150‒300 mAh g^−1^)^34, 36^, whereas the most stable (1 × 1) tunneled β-MnO_2_ phase^40, 41^ hardly incorporates Zn^2+^ ions^33^ due to narrow tunnels^42^. Furthermore, in the widely investigated ZnSO_4_ electrolyte, MnO_2_ generally suffers from capacity loss due to the dissolution of Mn^2+^ from Mn^3+^ disproportionation^34, 35^. Pre-addition of Mn^2+^ salt is proposed to improve capacity retention^35^ but the underneath mechanism remains unclear. Our previous study indicates that the use of zinc salt with bulky anion (e.g., CF_3_SO_3_^−^) benefits reactivity and stability of Zn anode and spinel ZnMn_2_O_4_ cathode^24^. Therefore, elucidating the electrode reactions of MnO_2_ and exploiting compatible electrolyte are desirable in developing rechargeable aqueous Zn-MnO_2_ batteries.

Herein, we report high-performance rechargeable aqueous Zn-MnO_2_ cells based on MnO_2_ cathode, Zn anode, and Zn(CF_3_SO_3_)_2_ electrolyte with Mn(CF_3_SO_3_)_2_ additive. For the widely investigated α-, β-, and γ-MnO_2_ polymorphs, we elucidate a common electrode reaction mechanism, by combining electrochemical measurements, X-ray diffraction analysis (XRD), elemental analysis, transmission electron microscopy (TEM), and synchrotron X-ray absorption spectroscopy (XAS). Interestingly, in the exemplified β-MnO_2_ that has been previously demonstrated unfavorable for Zn intercalation, a layer-type phase (i.e., Zn-buserite B-Zn_*x*_MnO_2_·*n*H_2_O) is generated during the initial discharge, followed by reversible insertion/extraction of Zn^2+^ ions in the layered structure (Fig. 1b). Up to ~ 0.5 Zn per molecular MnO_2_ is accommodated on discharging, along with disproportionated Mn dissolution and capacity fade. We significantly improve the cycling stability of Zn-MnO_2_ cell by employing concentrated Zn(CF_3_SO_3_)_2_ electrolyte and Mn(CF_3_SO_3_)_2_ additive (Fig. 1c). The pre-added Mn(CF_3_SO_3_)_2_ is found to suppress Mn^2+^ dissolution and result in the formation of a uniform porous MnO_*x*_ nanosheet layer on the cathode surface, which helps to maintain the electrode integrity. Remarkably, β-MnO_2_ exhibits high reversible capacity, high rate capability, and stable cyclability. We further demonstate a soft-packed Zn-MnO_2_ full cell that delivers a reversible capacity of 1550 mAh with a total energy density of 75.2 Wh kg^−1^ after 50 cycles.

## Results

### Materials synthesis and characterization

We selected pyrolusite β-MnO_2_ as a model polymorph, which has been previously demonstrated to exhibit extremely poor electrochemical activity^33^ and was prepared by a simple hydrothermal route in this study (detailedly described in experimental section). X-ray diffraction patterns (XRD, Supplementary Fig. 1a) reveals high purity of the formed tetragonal phase (JCPDS no. 24-735) with P42/mnm space group. Scanning electron microscope (SEM, Supplementary Fig. 1b) of the sample displays nanorod morphology with average length of 2 μm and width of 100–200 nm. Polymorphs of α-MnO_2_ and γ-MnO_2_ nanorods were also synthesized via hydrothermal technique (Supplementary Fig. 2; Supplementary Methods). Commerical β-MnO_2_ powders with large particle size of ~ 2 μm (Supplementary Fig. 3) were employed for comparison.

### Electrode reaction mechanism

**Fig. 2:**
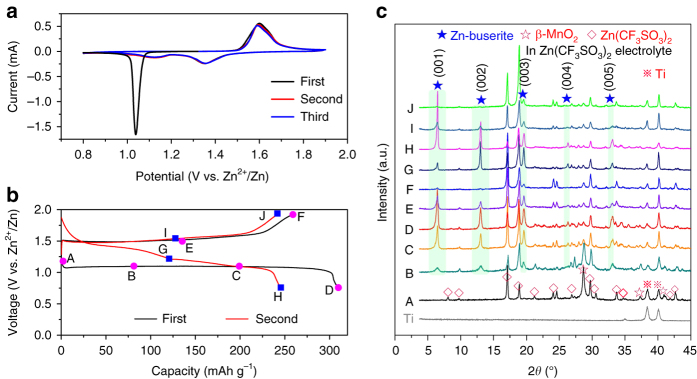
Electrochemical and structural evolution of β-MnO_2_ in Zn-MnO_2_ cell. **a** Cyclic voltammograms of β-MnO_2_ electrode at a scan rate of 0.1 mV s^−1^ from 0.8 to 1.9 V. **b** Typical charge/discharge curves for the initial two cycles at 0.32 C in 3 M Zn(CF_3_SO_3_)_2_ aqueous electrolyte. The points A–J marked the states where data were collected for XRD analysis. **c** XRD patterns of β-MnO_2_ electrode at selected states during the first and second cycles

Figure 2a shows the cyclic voltammograms (CVs) of β-MnO_2_ in aqueous 3 M Zn(CF_3_SO_3_)_2_ electrolyte. A sharp peak at around 1.06 V is observed during the first cathodic sweeping. In the following cycles, the CV curves are well repeated with two cathodic peaks located at 1.35 and 1.17 V and an overlapped anodic peak at 1.6/1.65 V. The significant difference in CV profiles between the initial and subsequent cycles suggests phase transition. Figure 2b shows the typical galvanostatic profiles of β-MnO_2_ at 0.32 C. The first discharge curve displays a flat plateau at around 1.08 V while the second cycle presents two slopping discharge plateaus, in line with the CV results. Notably, the initial discharge capacity reaches 307 mAh g^−1^, which approaches the theoretical capacity of 308 mAh g^−1^ (based on MnO_2_) and corresponds to 0.5 Zn^2+^ per MnO_2_. The evolution of CV profiles and discharge plateaus indicates different mechanism of Zn^2+^ intercalation in MnO_2_ electrode^36, 43^, as discussed below.

To probe the structural evolution of β-MnO_2_ in the discharge/charge process, ex-situ XRD patterns (Fig. 2c) were recorded at the selected states (marked points in Fig. 2b). On first discharging (A → D), the characteristic peaks of β-MnO_2_ are gradually weakened and new phase arises. Besides the peaks designated to the Zn(CF_3_SO_3_)_2_ salt, new peaks emerge at 6.47, 13.00, 19.58, 26.28, and 32.93°, which could be assigned to reflections from the (001)–(005) crystallographic planes of a layered Zn-buserite phase, respectively. The electrolyte salt is precipitated on the surface of both MnO_2_ cathode and Zn anode but can be easily removed by immersing and rinsing with water (Supplementary Fig. 4a–c). Notably, the XRD pattern of the rinsed cathode differs from that of previously reported species (e.g., MnOOH^35^, spinel ZnMn_2_O_4_^33^, birnessite^38^, tunneled γ-ZnMnO_2_^34^, and layered L-Zn_*x*_MnO_2_^34^) in discharged MnO_2_ electrodes (Supplementary Fig. 4d). Rietveld refinement of the XRD data of the discharged electrode suggests the formation of Zn-buserite phase (Supplementary Fig. 4e). The exact structural motif of Zn-buserite is not determined yet, but will be further investigated in the future. The Zn-buserite phase, commonly found in layered Mn oxide mineral^44–46^, contains H_2_O layers in the channels between two MnO_6_ octohedron slabs (Fig. 1b), featuring a similar structure with Ca-buserite^44^ (JCPDS No.50-0015). Zn^2+^ cations reside above and below the Mn vacant sites and are coordinated with three O atoms adjacent to the vacancies and three O atoms from interlayer H_2_O^36, 44–46^. The presence of H_2_O in the discharged species was validated using thermal gravimetric analysis (TGA), indicating a composition of ~ 2.28 molecular H_2_O per formula of Zn-buserite (Supplementary Fig. 5). In the followed charging process (D→F), the intensity of characteristic peaks for the layered phase was gradually weakened upon extraction of Zn ions. This peak attenuation could be explained by the decrease of scattering atom concentration in unit cell and the weakening of Zn–O interaction due to Zn egress. Similar intensity variation of (00*l*) reflection has been observed on layered intercalation electrodes such as vanadium oxides^9, 47^. In the second cycle, the signals of layered compound were reversibly strengthened/weakened upon Zn^2+^ insertion/extraction. The presence of β-MnO_2_ can be observed in the initial several cycles but is not discernable after 10 cycles (Supplementary Fig. 6). We investigated the structural evolution of α-MnO_2_ and γ-MnO_2_ cathodes as well. Interestingly, these two polymorphs undergo phase transformation to layered Zn-buserite upon first discharging and reversible Zn intercalation in the layered structure on subsequent cycling (Supplementary Figs. 7 and 8), resembling the case of β-MnO_2_. The results suggest common electrode reaction mechanism in tunneled polymorphs of MnO_2_, which to the best of our knowlege, is first elucidated in mild acidic electrolytes.

**Fig. 3:**
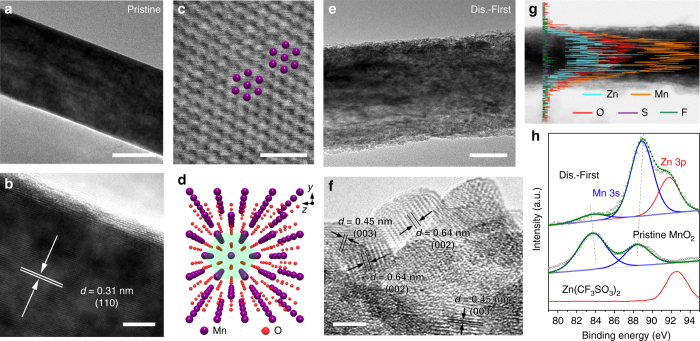
Microstructural and compositional analysis of MnO_2_. **a** TEM image, **b** HRTEM image, **c** ABF-STEM image, and **d** schematic atomic model (viewed from the [100] zone axis) at the initial state. **e** TEM image, **f** HRTEM image, **g** EDS line scanning profiles in TEM, and **h** XPS spectra of the first fully discharged electrode. *Scale bars*, 50 nm **a**, **e**; 5 nm **b**, **f**; and 1 nm **c**, respectively

The structural evolution of β-MnO_2_ electrode was further investigated by ex-situ TEM analysis. Figure 3a, b displays the TEM and high-resolution TEM (HRTEM) images at the initial state, where the lattice fringes can be indexed to the (110) plane of β-MnO_2_. The annular bright field-scanning TEM (ABF-STEM) image (Fig. 3c) clearly shows the atomic arrangement within the tunnel-like framework, as schematically viewed along the [100] direction of the lattice (Fig. 3d). After fully discharging, the one-dimensional nanorod shape is maintained, while the surface of electrode becomes rough with the formation of aggregated nanoparticles (Fig. 3e), which is ascribed to the structural distortion in the phase-conversion process. The observed lattice fringes with interplanar distances of 0.45, 0.64, and 1.29 nm correspond to the (003), (002), and (001) planes of Zn-buserite (Fig. 3f and Supplementary Fig. 9a), respectively, consistent with the XRD analysis.

To eliminate the impact of precipitated electrolyte salt, the discharged electrode was rinsed with water for elemental dispersive spectroscopy (EDS) and X-ray photoelectron spectroscopy (XPS) analysis. The line scanning profile in TEM (Fig. 3g) and elemental mapping (Supplementary Fig. 9b) in STEM of the discharged electrode reveal the uniform distribution of Zn, Mn and O, whereas S and F from electrolyte are not detectable (Fig. 3g). In XPS spectra, the energy splitting (Δ*E*) of Mn 3 s doublet peaks is 4.7 and 5.0 eV for pristine and discharged electrodes, respectively, indicating reduced Mn valence after Zn insertion (Fig. 3h). At discharged state, a new Zn 3p peak appears at 92.0 eV, which is lower than that of Zn(CF_3_SO_3_)_2_ (92.7 eV) and could be assigned to the intercalated Zn. These results confirm the presence of Zn^2+^ ions into the layered manganese oxide host and rule out the possibility of electrode reactions associated with CF_3_SO_3_^−^ anions. Furthermore, the TEM images of β-MnO_2_ electrode after different cycles (Supplementary Fig. 10) indicate expansion and exfoliation of nanorods, which is attributed to the phase transition, Mn dissolution and repeated Zn^2+^ intercalation, and would incur capacity loss during cycling.

**Fig. 4:**
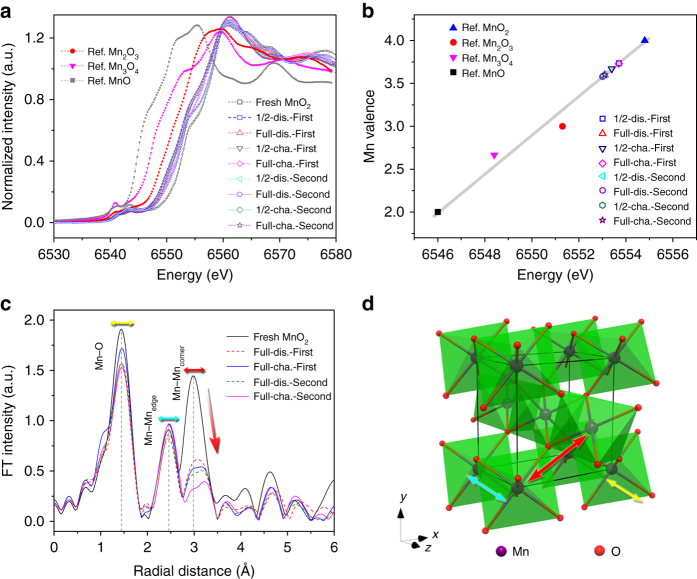
XAS characterization of β-MnO_2_ electrode. **a** Mn-K edge XANES curves at selected discharge/charge states, with reference to standard MnO, Mn_2_O_3,_ and Mn_3_O_4_. **b** Fitted linear relationship between the photon energy and oxidation state of Mn element. **c** The EXAFS spectra. **d** Schematic depiction of the unit cell of β-MnO_2_

To gain insight into the variation of Mn oxidation state and electronic structure during the (de)intercalation process, we performed the synchrotron XAS characterization, which has been demonstrated useful to analyze manganese oxides^48–52^. Figure 4a shows the normalized Mn K-edge XANES (X-ray near edge absorption structure) profiles of β-MnO_2_ electrode at selected states in the initial two cycles. The nominal Mn valence was plotted vs. excitation energy of reference manganese oxides to establish fitted linear correlation (Fig. 4b). On discharging, the entire edge shifts toward lower energy, indicating a decrease of the average Mn oxidation state. The mean Mn valence at fully discharged state is estimated to be 3.6. During first charging, the edge position slightly shifts back to higher energy, while it remains almost unchanged in the second cycle. The interesting point is that the Mn valence should increase/decrease with Zn^2+^ intercalation/deintercalation and would approach 3 for the fully discharged electrode, as anticipated from the discharged capacity (Fig. 2b). We postulate that such unexpected observation could be ascribed to the disproportional dissolution of trivalent Mn species (Mn^3+^_s_ → Mn^4+^_s_ + Mn^2+^_aq_)^38, 53^. Analysis of Mn by inductively coupled plasma atomic emission spectrometer (ICP-AES) evidences the change of Mn concentration in the electrolyte (Supplementary Fig. 11; Supplementary Note 1). On discharging, the amount of dissolved Mn increases and corresponds to ~ 8.9% of the total manganese at full discharge. The partial dissolution of Mn in electrolyte is a feasible attribution to the noticeable capacity loss on cycling.

Figure 4c shows the EXAFS (extended X-ray absorption fine structure) spectra of β-MnO_2_ electrode at selected Zn (de)intercalation stages. The strongest peak located at 1.5 Å is attributed to the closest oxygen (Mn-O) in the MnO_6_ octahedra. The peaks at 2.5 and 3.0 Å are assigned to Mn in the edge-sharing (Mn-Mn_edge_) and corner-sharing (Mn-Mn_corner_) MnO_6_ octahedra (Fig. 4d), respectively^51, 54^. When the elcctrode was fully discharged, the relative intensity of the Mn-Mn_corner_ peak decreased to a much larger extent than that of Mn-O and Mn-Mn_edge_ signals (Supplementary Fig. 12). This result is indicative of the breakage of the corner-shared MnO_6_ octahedra. Furthermore, the 3.0 Å peak broadens and slightly shifts to larger distance, which is related to the formation of Mn-O-Zn energy-absorbing path between the layered MnO_6_ octohedron slabs and inserted Zn ions. A comparison of the crystallographic structure between β-MnO_2_ and Zn-buserite suggests that the co-insertion of Zn^2+^ and H_2_O and the dissolution of Mn distort the pyrolusite framework, leaving Mn vacancies in the upper/underlying layers and generating layered Zn-buserite. This tunnel-to-layer phase transition is irreversible, as indicated by the absence of EXAFS spectra recovery on first recharge. Meanwhile, the broadened 3.0 Å peak is not fully recovered after second charging, which can be attritubed to the capacity loss (Fig. 2b). Notably, XAS analysis of α-MnO_2_ and γ-MnO_2_ electrodes (Supplementary Fig. 13) reveals similar behavior with that of β-MnO_2_, again suggesting common electrode reaction mechanism among different tunneled MnO_2_ polymorphs.

### Electrochemical performance

To evaluate the electrochemical performance, coin-type Zn-MnO_2_ cell was assembled in ambient air by using β-MnO_2_ nanorod cathode, Zn foil anode, filter paper separator, and aqueous Zn(CF_3_SO_3_)_2_ electrolyte. The concentrated 3 M Zn(CF_3_SO_3_)_2_ results in better cyclic stability than diluted electrolyte (e.g., 1 M) (Supplementary Fig. 14), which is ascribed to the decrease of water activity and water-induced side reactions^4, 24, 55^. As shown in Fig. 1c, the cells based on mild acidic electrolye (3 M ZnSO_4_, pH ~ 3.4; 3 M Zn(CF_3_SO_3_)_2_, pH ~ 3.6) show much better cycling performance as compared with that employing KOH electolyte. Meanwhile, the cell using Zn(CF_3_SO_3_)_2_ electrolyte delivers much higher initial discharge capacity than that of ZnSO_4_ (275 vs. 120 mAh g^−1^) at 0.65 C. However, similar capacity deterioration is observed upon cycling, due to the loss of active mass. To address this issue, we pre-added Mn^2+^ salts into the electrolyte to accommodate the dissolution equilibrium of Mn^2+^ from MnO_2_ electrode. By eliminating the anion effect, we selected Mn(CF_3_SO_3_)_2_ as the electrolyte additive, with concentration from diluted 0.01 M to the saturated 0.1 M. The optimized electrolyte composition was found to be 3 M Zn(CF_3_SO_3_)_2_ + 0.1 M Mn(CF_3_SO_3_)_2_, which results in the highest Coulombic efficiency and ionic conductivity as well as high capacity of 225 mAh g^−1^ after 100 cycles (Supplementary Figs. 15 and 16).

**Fig. 5:**
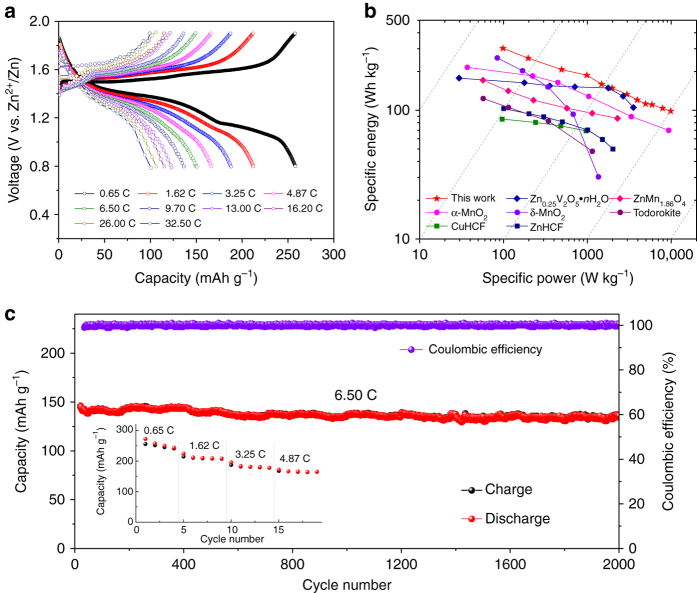
Electrochemical performance of Zn-MnO_2_ cells in 3 M Zn(CF_3_SO_3_)_2_ electrolyte with 0.1 M Mn(CF_3_SO_3_)_2_ additive. **a** Discharge/charge profiles at varying C rates. **b** The Ragone plots of Zn-MnO_2_ battery and ZIBs with other reported cathode materials. Values are based on the total active mass of both cathode and anode. **c** Long-cycle performance at rate of 6.5 C. *Inset* shows the capacity evolution at the initial 19 cycles

Figure 5a shows the charge/discharge profiles of Zn-MnO_2_ cells at different current densities. Discharge capacities of 258, 213, 188, 151, and 115 mAh g^−1^ were recorded at rates of 0.65, 1.62, 3.25, 6.50, and 16.20 C, respectively. Even at a high rate of 32.50 C, a reversible capacity of 100 mAh g^−1^ could be obtained. In addition, when the rate shifted back to 0.65 C, the capacity recovered to 246 mAh g^−1^, showing a strong tolerance to the rapid Zn^2+^ ions insertion/extraction (Supplementary Fig. 17). The superior rate performance can be further viewed from the Ragone plots (specific energy vs. specific power) by comparing the Zn-MnO_2_ system to reported α-MnO_2_^33^, δ-MnO_2_^56^, Zn_0.25_V_2_O_5_·nH_2_O^9^, Zn_1.86_Mn_2_O_4_^24^, todorokite^37^, KCuFe(CN)_6_ (CuHCF)^21^, and Zn_3_[Fe(CN)_6_]_2_ (ZnHCF)^57^ cathodes for aqueous ZIBs (Fig. 5b). High-specific energy and specific power (254 Wh kg^−1^ at 197 W kg^−1^; 110 Wh kg^−1^ at 5910 W kg^−1^) can be simultaneously achieved, which is promising for energy storage applications. The Zn-MnO_2_ cell was galvanostatically discharged/charged at 6.50 C (Fig. 5c) to evaluate the long-term cycling stability. Remarkably, the reversible capacity sustains 135 mAh g^−1^ with a capacity retention of 94% over 2000 cycles and Coulombic efficiency approaching 100%.

We also investigated the Zn-MnO_2_ cells with 3 M ZnSO_4_ + 0.1 M MnSO_4_ and 3 M Zn(CF_3_SO_3_)_2_ + 0.1 M MnSO_4_ electrolytes, which delivered initial discharge capacity of 110 and 205 mAh g^−1^, respectively (Supplementary Fig. 18). In SO_4_^2−^-based electrolyte, an increase of capacity was observed within the first several cycles, which was attributed to the activation process that has been similarly found in reported α-/γ-MnO_2_ cathodes^34–36^. Interestingly, the CF_3_SO_3_^−^-based electrolyte endows much higher initial discharge capacity (275 mAh g^−1^ at 0.65 C) and results in capacity stabilization after ~ 10 cycles. The different behaviors could be ascribed to the Zn(CF_3_SO_3_)_2_ solution that not only features higher ionic conductivity (Supplementary Fig. 19) but also enables faster kinetics and higher stability of Zn plating/stripping as compared with sulfate and alkaline electrolytes (Supplementary Fig. 20; Supplementary Note 2). Besides, the bulky CF_3_SO_3_^−^ anion (vs. SO_4_^2−^ with double charge) could decrease the number of water molecules surrounding Zn^2+^ cations and reduce the solvation effect^24^, thus facilitating Zn^2+^ ions transportation and charge transfer.

Although the pre-addition of Mn^2+^ in electrolyte has been demonstrated to enhance the cyclability of MnO_2_ electrode^35^, the underneath mechanism remains unclear. To further understand the functions of pre-added Mn^2+^, we have carried out a series of analytical studies, including electrochemical measurements, XRD, Raman, XPS, XANES, and SEM/TEM. In acidic electrolyte, manganese oxides (MnO_*x*_) such as MnO_2_ or Mn_2_O_3_ can be generated from electrolysis of Mn^2+^-containing solution^32^ based on the following reactions:

1 $${\rm{Mn}}{{\rm{O}}_2} + 4{{\rm{H}}^ + } + 2{{e}^ - } = {\rm{M}}{{\rm{n}}^{2 + }} + 2{{\rm{H}}_2}{\rm{O}}\quad \\ {E^{\rm{\theta }}} = 2.01\,{\rm V}\left( {{\rm{vs}}.\,\;{\rm{Z}}{{\rm{n}}^{2 + }}/{\rm{Zn}}} \right)$$

2 $${\rm{M}}{{\rm{n}}_2}{{\rm{O}}_3} + 6{{\rm{H}}^ + } + 2{{\rm{e}}^ - } = 2{\rm{M}}{{\rm{n}}^{2 + }} + 3{{\rm{H}}_2}{\rm{O}}\quad \\ {E^{\rm{\theta }}} = 2.26\,{\rm V}\left( {{\rm{vs}}.\,\;{\rm{Z}}{{\rm{n}}^{2 + }}/{\rm{Zn}}} \right)$$

According to Nernst equation, the required theoretical potentials to form MnO_2_ and Mn_2_O_3_ in Mn(CF_3_SO_3_)_2_ (0.1 M, pH 6.0) solution are 1.35 and 1.26 V (vs. Zn^2+^/Zn), respectively; the corresponding values are 1.60 and 1.64 V in 3 M Zn(CF_3_SO_3_)_2_ electrolyte containing 0.1 M Mn^2+^ (pH 3.8). This estimation is consistent with the voltammetry results of three-electrode measurements (Supplementary Fig. 21; Supplementary Note 3), which also reveals that Mn^2+^ is not reduced within the investigated potential windows. After charging in 3 M Zn(CF_3_SO_3_)_2_ + 0.1 M Mn(CF_3_SO_3_)_2_ electrolyte, brown deposit layer was observed on the electrode. The layer is composed of manganese oxide with Mn oxidation state between + 3 and + 4, and features nanosheet morphology and poor crystallinity, as analyzed by SEM, XRD, Raman, XPS, and XAENS (Supplementary Fig. 22).

**Fig. 6:**
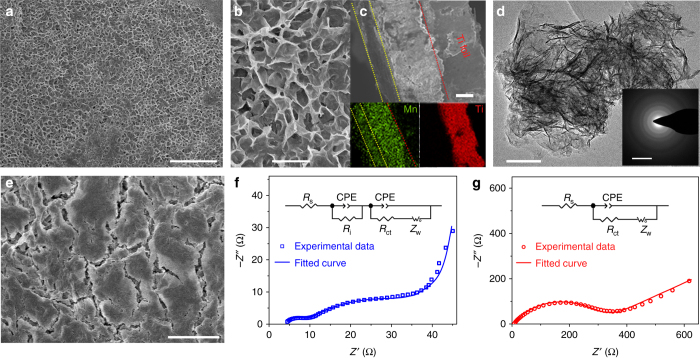
Function of pre-added Mn^2+^ in electrolyte. **a**, **b**, **c**, **e** SEM, **d** TEM images, and **f**, **g** three-electrode-cell EIS analysis of re-obtained cathodes after ten cycles in 3 M Zn(CF_3_SO_3_)_2_ electrolyte **a**–**d**, **f** with and **e**, **g** without 0.1 M Mn(CF_3_SO_3_)_2_ additive. *Insets* of **c**, **d** show elemental mapping and SAED pattern, respectively. *Insets* of **f**, **g** show the equivalent circuit to fit the EIS data, where *R*_s_, *R*_i_, *R*_ct_, CPE, and *Z*_w_ represent series resistance, interface resistance between electrolyte and deposited layer, charge-transfer resistance, constant-phase element, and Warburg diffusion process, respectively. *Scale bars*, 5 μm **a**, **e**; 1 μm **b**; 10 μm **c**; 100 nm **d**; and 5 1/nm (*inset* of **d**), respectively

In post-mortem analysis of Zn-MnO_2_ cell using 3 M Zn(CF_3_SO_3_)_2_ + 0.1 M Mn(CF_3_SO_3_)_2_ electrolyte, we also observed an interconnected porous MnO_*x*_ layer on the cathode surface after charging (Fig. 6a, b). The cross-sectional SEM image and elemental mapping images (Fig. 6c) evidence the presence of a uniform layer with thickness around 10 μm. TEM imaging and selected area electron diffraction (SAED) analysis reveal porous nanosheet microstructure and amorphous character of the deposited layer (Fig. 6d), which would facilitate mass diffusion. In contrast, the integrity of β-MnO_2_ electrode was seriously destroyed with the formation of cracks in 3 M Zn(CF_3_SO_3_)_2_ electrolyte without Mn(CF_3_SO_3_)_2_ additive (Fig. 6e). The electrode pulverization would break the electronic conducting network and increase electrode polarization, further aggravating the capacity decay. Electrochemical impedance spectroscopy (EIS) was performed in a three-electrode cell, using the dismantled cathode after ten cycles as the working electrode, platinum plate as the counter electrode, and saturated calomel electrode (SCE) as the reference electrode. The cycled electrode in Mn^2+^-added electrolyte displays two depressed semicircles in high frequency area and one line in low frequency region (Fig. 6f). Fitting the EIS data (Supplementary Table 1) gives a series resistance (*R*_s_, 4.5 Ω), an interface resistance (*R*_i_, 6.0 Ω) between electrolyte and deposited layer, a charge-transfer resistance (*R*_ct_, 25 Ω) and a Warburg diffusion impedance (*Z*_w_, 124.7 Ω). In comparison, the cell without electrolyte additive shows higher *R*_s_ (8.0 Ω), *R*_ct_ (350 Ω), and *Z*_w_ (1200 Ω), in the absence of the apparent interface component (Fig. 6g).

Based on the above results, we propose three merits of the Mn^2+^ electrolyte additive for the Zn-MnO_2_ battery: (1) accommodating and compensating Mn^2+^ dissolution from the electrode, (2) improving initial Coulombic efficiency and ionic conductivity of the electrolyte and (3) generating a uniform porous nanostructured MnO_*x*_ film on the cathode surface, which helps to maintain the electrode integrity and favor charge transfer. Note that the generated MnO_*x*_ layer itself contributes to nearly 2.4% of the capacity delivered by the active material (Supplementary Fig. 23). The Zn(CF_3_SO_3_)_2_ + Mn(CF_3_SO_3_)_2_ electrolyte is also applicable to improve the cycling stability of nanostructured α-MnO_2_ and γ-MnO_2_ cathodes (Supplementary Fig. 24a, b). Furthermore, commercial β-MnO_2_ powders with irregular shape and micrometer particle size (Supplementary Fig. 3) also exhibit considerable capacity (132 mAh g^−1^ at 0.65 C) and cyclability (200 cycles) in this electrolyte (Supplementary Fig. 24c, d).

The Zn anode was also investigated to understand the high-performing Zn-MnO_2_ cell chemistry (Supplementary Figs. 25–28). Post-mortem analysis of cycled Zn in three-electrode cell with 3 M Zn(CF_3_SO_3_)_2_ + 0.1 M Mn(CF_3_SO_3_)_2_ electrolyte reveals a dense and dendrite-free surface morphology after 280 h of repeated Zn plating/stripping (Supplementary Fig. 25). In a Zn-MnO_2_ full cell, neither dendritic morphology nor formation of byproducts such as ZnO or Zn(OH)_2_ was evidenced after rate test (Supplementary Fig. 26), favoring the cyclic stability of Zn-MnO_2_ batteries. In contrast, in 3 M ZnSO_4_ + 0.1 M MnSO_4_ electrolyte, Zn plate with lots of cracks formed on the zinc surface, while ZnO nanorods were observed in KOH electrolyte, which would deter the cyclability of Zn (Supplementary Figs. 25 and 27). Furthermore, the EDS analysis indicates that there is no detectable Mn in Zn anode (Supplementary Fig. 28).

## Discussion

**Fig. 7:**
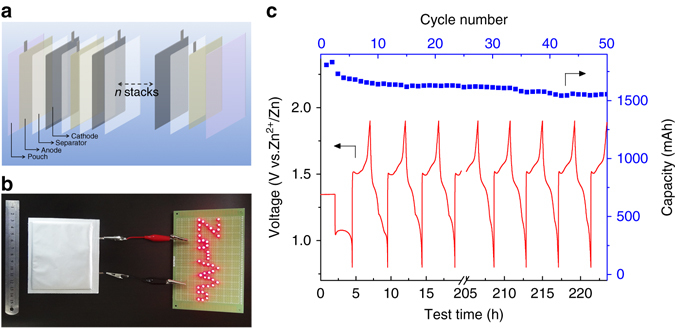
Electrochemical performance of pouch-type Zn-MnO_2_ battery. **a** Schematic illustration of the cell configuration with anode—separator—cathode stacks. **b** A digital photo of the soft-package battery powering a series of LED lights. **c** Cycling performance in the voltage range of 0.8–1.9 V at constant current of 0.72 A

The exceptional performance of Zn-MnO_2_ coin-type batteries has motivated us to further assess soft-packed full cells, which were facilely assembled in ambient air negating complicated procedures or extra protection (Supplementary Methods). Figure 7a schematically shows the battery configuration consisting of six anode—separator—cathode stacks. A typical assembled pouch-type cell lightens a “Zn-Mn”—shape indicator containing 44 LEDs (Fig. 7b). A stable discharge capacity of 1550 mAh can be obtained after 50 repeated cycles with an average potential of 1.35 V (Fig. 7c). The full cell delivers an energy density of 158.5 Wh kg^−1^ based on the total weight of the active materials (including both cathode and anode). This value far exceeds that of other aqueous Li-ion batteries (50–90 Wh kg^−1^)^3, 4, 58^ and aqueous Na-ion batteries (~ 33 Wh kg^−1^)^8, 16, 59^. Remarkably, a total energy density of 75.2 Wh kg^−1^ is obtained according to the mass of whole battery mass, much higher than that of commercial Pb-acid (~ 30 Wh kg^−1^) and Ni-Cd technologies (~ 50 Wh kg^−1^)^26^. We note that the higher price of anhydrous Zn(CF_3_SO_3_)_2_ salt relative to ZnSO_4_ and KOH would inevitably increase the practical cost of this aqueous Zn-MnO_2_ battery system, even though Zn(CF_3_SO_3_)_2_ merely serves as charge carrier and is not consumed during battery operation. Fortunately, considering the abundant, cheap precursors (i.e., triflic acid and ZnCO_3_)^60^ and the direct usage of hydrate-form salt in aqueous solution, the cost of Zn(CF_3_SO_3_)_2_ electrolyte could be expected to drop with the development of synthetic technique and market demand.

In conclusion, we demonstrate a high-performing rechargeable Zn-MnO_2_ battery system based on zinc anode, β-MnO_2_ cathode, and mild acidic aqueous electrolyte. We elucidate the Zn-insertion mechanism and structural evolution of MnO_2_ cathode by combining electrochemical investigations, XRD, TEM, ICP, and XANES/EXAFS analysis. A phase transition from tunneled to layered structure (Zn-buserite) occurs during the first discharge of MnO_2_, followed by reversible Zn^2+^ (de)intercalation in the H_2_O-containing Zn-buserite framework. Unlike previous reports, this electrode mechanism is common in polymorphs of α-MnO_2_, γ-MnO_2_, and β-MnO_2_. The phase tranformation, Mn dissolution and electode pulverization incur capacity fade of MnO_2_. By formulating an aqueous 3 M Zn(CF_3_SO_3_)_2_ + 0.1 M Mn(CF_3_SO_3_)_2_ electrolyte, the Mn^2+^ dissolution can be effectively accommodated and the electrode integrity can be maintained because of the in-situ generated amorphous MnO_*x*_ layer. As a result, Zn-MnO_2_ cell exhibits high capacity (225 mAh g^−1^ at 0.65 C), high rate capability (100 mAh g^−1^ at 32.50 C) and long-term cycling stability (94% capacity retention after 2000 cycles at 6.50 C). Furthermore, the assembled soft-packed Zn-MnO_2_ battery can deliver a high reversible capacity of 1550 mAh with a total energy density of 75.2 Wh kg^−1^, among the highest value achieved in aqueous battery technologies. The present Zn-MnO_2_ system holds great promise for potential applications in large-scale energy storage, in view of the remarkable electrochemical performance and other advantages such as low materials cost, easy manufacturing, high safety, and environmental friendliness.

## Methods

### Synthesis

β-MnO_2_ nanorods were synthesized by a hydrothermal method. In a typical synthesis, 30 ml KMnO_4_ (0.1 M) and 30 ml MnSO_4_·H_2_O (0.6 M) were mixed under continuous stirring for 30 min at room temperature. The mixture was loaded into a 100 ml Teflon-lined autoclave and maintained at 140 °C for 12 h. The obtained product was centrifuged, washed thoroughly using water and absolute ethyl alcohol, and dried at 80 °C for 10 h. Bulk β-MnO_2_ powders was purchased from *Alfa Aesar*. α-MnO_2_ and γ-MnO_2_ nanorods were synthesized via hydrothermal technique following previously reported procedures^61^.

### Characterization

Powder XRD patterns were collected on a Rigaku X-ray diffractometer (MiniFlex600) with Cu Kα radiation. SEM images were obtained on Field-emission JEOL JSM-7500F microscope. TEM and HRTEM images were taken on Philips Tecnai G2 F20. ABF-STEM was performed on Titan Cubed Themis G2 300 (FEI) at an acceleration voltage of 200 kV. The XAS data were collected on BL14W1 beamline of Shanghai Synchrotron Radiation Facility and analyzed with software of Ifeffit Athena^62^. ICP-AES measurements were conducted on a PerkinElmer Optima 8300. XPS was tested on a Perkin Elmer PHI 1600 ESCA system. Raman spectra were obtained on confocal Thermo-Fisher Scientific DXR microscope using 532 nm excitation. TGA was measured by a Netzsch STA 449 F3 Jupiter analyzer.

### Electrochemical test

Electrochemical performance was tested using CR2032 coin-type cells. The working electrode was fabricated by blending MnO_2_ powder, Super P carbon and polyvinylidene fluoride in a weight ratio of 8:1:1 using *N*-methyl-2-pyrrolidone as solvent. The obtained slurry was pasted onto a Ti foil and vacuum-dried at 100 °C for 12 h. The loading mass of active material was ~ 2 mg cm^−2^. Filter paper and zinc foil were employed as the separator and anode, respectively. A 3 M Zn(CF_3_SO_3_)_2_ with/without 0.1 M Mn(CF_3_SO_3_)_2_ aqueous solution was used as the electrolyte. The assembled cells were galvanostatically cycled between 0.8 and 1.9 V using the LAND-CT2001A battery-testing instrument. Calculation of specific capacities was based on the mass of initial MnO_2_. CVs were measured on a Parstat 263 A electrochemical workstation (AMETEK). EIS was performed on a Parstat 2273 electrochemical workstation (AMETEK). The AC perturbation signal was ± 10 mV and the frequency ranged from 100 kHz to 100 mHz. The electrochemical behaviors of Mn^2+^ additive in electrolyte were characterized using three-electrode cells (Ti foil as working electrode, platinum plate or Zn foil as counter electrode, and SCE as reference electrode).

### Data availability

The authors declare that all the relevant data are available within the paper and its Supplementary Information file or from the corresponding author upon reasonable request.

## Electronic supplementary material

Supplementary Information
