# Supplementary material for: Rechargeable aqueous zinc-manganese dioxide batteries with high energy and power densities
Source: Nat Commun. 2017 Sep 1;8:405. doi: 10.1038/s41467-017-00467-x (PMC5581336; doi:10.1038/s41467-017-00467-x)
Supplement: Supplementary file 1 — Supplementary Information [file 41467_2017_467_MOESM1_ESM.pdf]

File name: Supplementary Information
Description: Supplementary figures, supplementary table 1, supplementary notes, supplementary methods and supplementary references.

Supplementary informantion

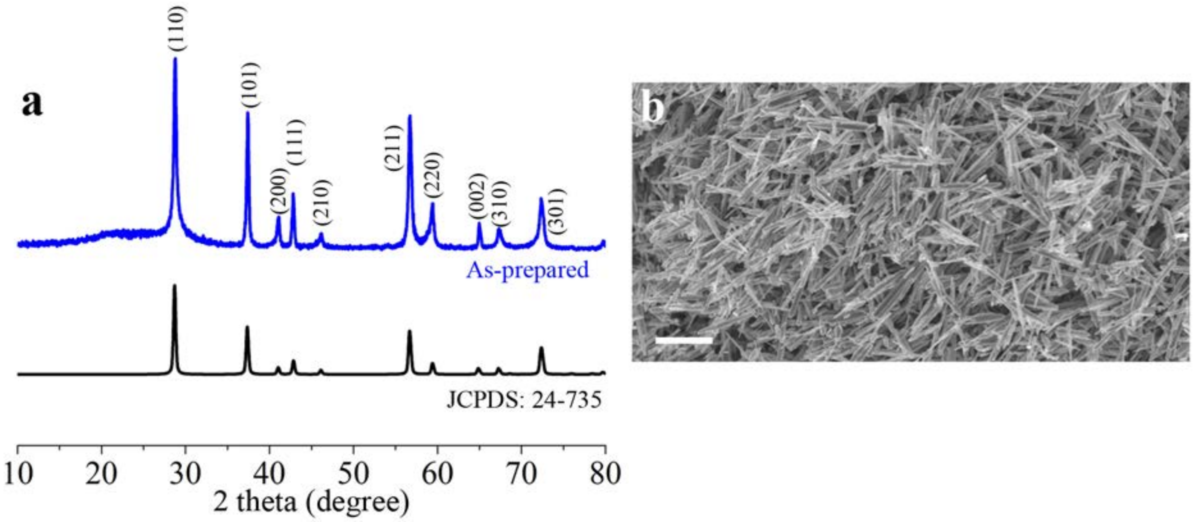

Supplementary Figure 1 | Phase and morphology characterization of Pyrolusite β-MnO_2. (**a**) XRD pattern, (**b**) SEM image of as-prepared MnO_2. Scale bar, 2 μm.

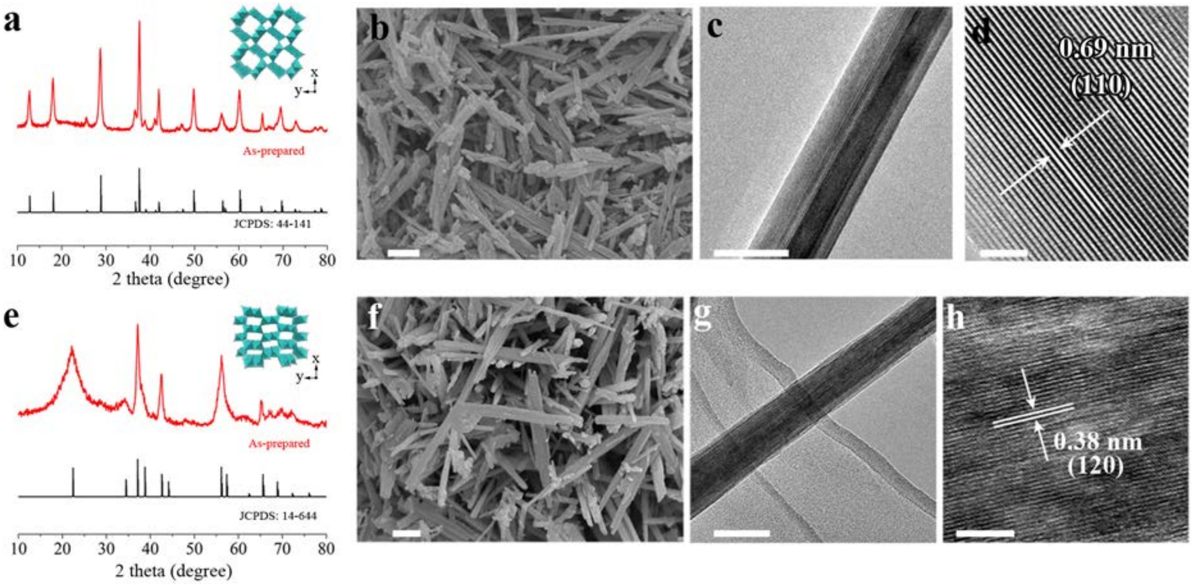

Supplementary Figure 2 | Characterization of α-MnO_2 and γ-MnO_2. (**a,e**) XRD patterns, (**b,f**) SEM images, (**c,g**) TEM and (**d,h**) HRTEM images of (**a-d**) as-prepared α-MnO_2 and (**e-h**) as-prepared γ-MnO_2. Supplementary Fig. 2a,e insets show the schematic structures of α-MnO_2 and γ-MnO_2, respectively. Scale bars, 200 nm (**b,f**), 50 nm (**c,g**) and 5 nm (**d,h**), respectively.

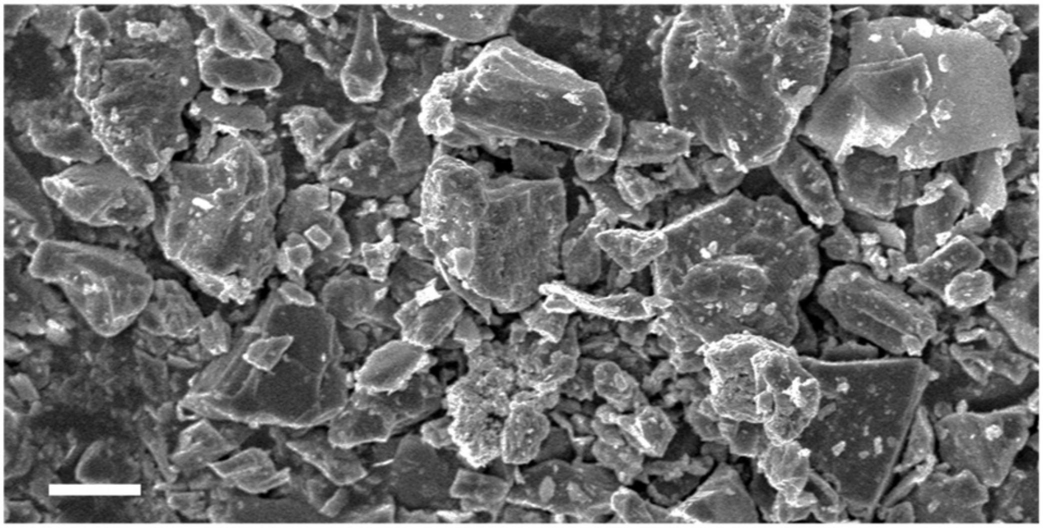

Supplementary Figure 3 | SEM image of commercial β-MnO_2. Scale bar, 2 μm.

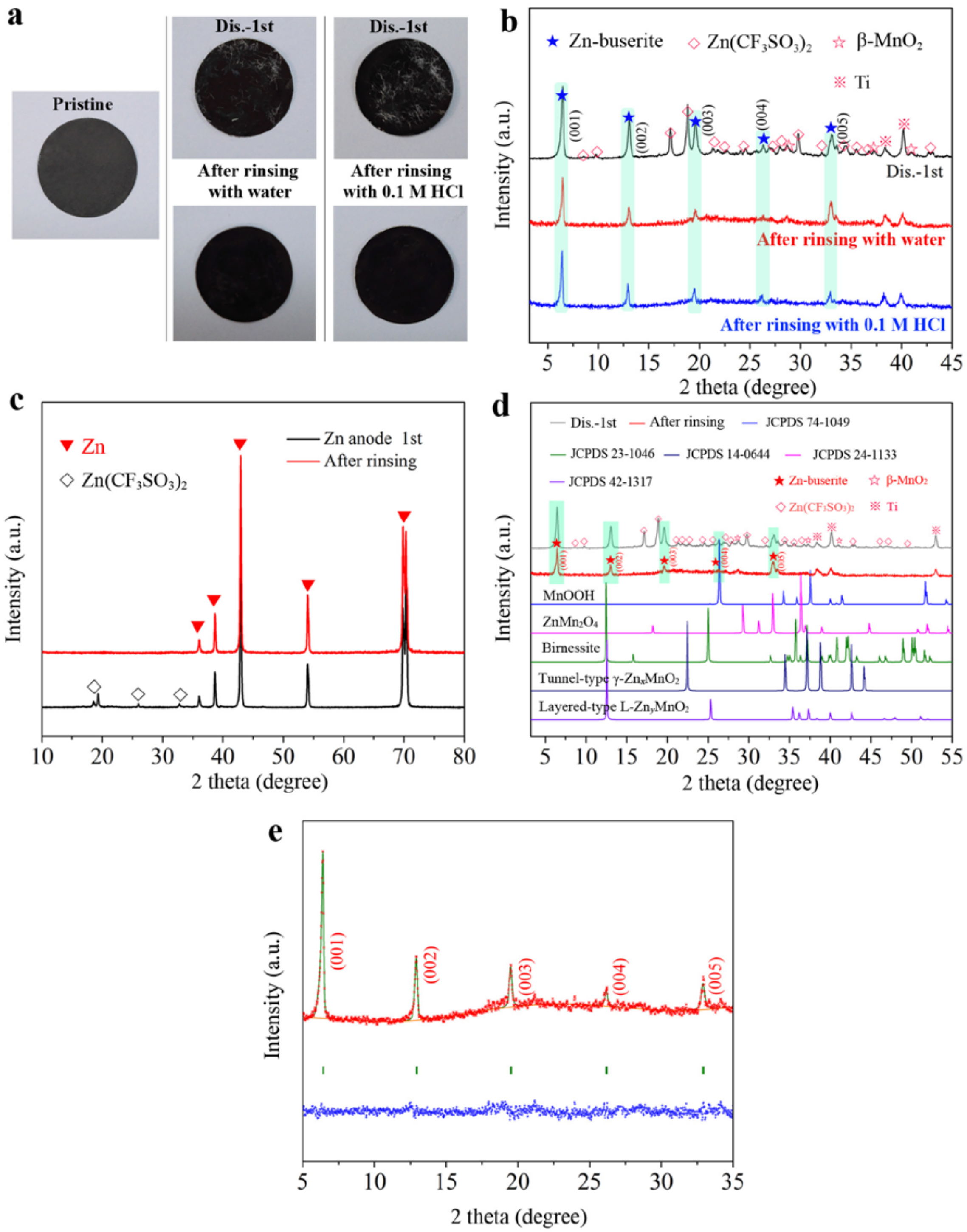

Supplementary Figure 4 | Analysis of discharged species in cathode and characterization of Zn anode. (**a**) Digital photos of pristine, discharged and rinsed cathodes with water or 0.1 M HCl solution. (**b**) The corresponding XRD patterns of the original discharged cathode and rinsed cathode with water and 0.1 M HCl solution. (**c**) XRD patterns of cycled Zn anode before and after rinsing with water.

Obviously, the electrolyte salt on Zn surface can be easily rinsed. (**d**) The XRD patterns of the discharged β-MnO_2 cathode and the standard phases previously reported for the discharged product of MnO_2 cathode in $ZnSO_4$-based electrolyte. Clearly, the XRD pattern of the discharged electrode totally differs from the characteristic peaks of previously reported species, such as MnOOH[1], birnessite[2], spinel-type $ZnMn_2O_4$[3,4], tunnel-type γ-Zn_xMnO_2[4], and layered-type L-Zn_yMnO_2[4]. (**e**) Rietveld refined XRD pattern of the discharged electrode. Experimental data, calculated results, allowed Bragg diffraction positions and difference profile are marked with red dots, cyan line, vertical bars and blue circles, respectively. Space group: C2/m (no. 12). Cell parameter: a=5.004 Å, b=2.850 Å, c=16.082 Å. $\alpha=90^o$, $\beta=116.4^o$, $\gamma=90^o$. Rwp= 8.67%, Rp= 8.42%.

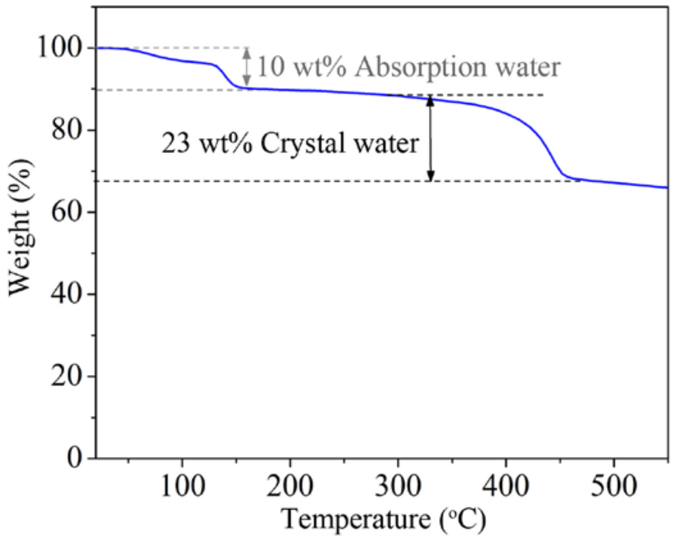

Supplementary Figure 5 | TG analysis. The water content in Zn-buserite after first discharge was obtained from TGA profiles in Ar atmosphere from 20 °C to 550 °C. The crystal water is around 25.55 wt% of the discharged product (23% ÷ 90% = 25.55%). Supposing the formula of $Zn_{0.5}MnO_2 \bullet nH_2O$ for Zn-buserite, *n* is around 2.28.

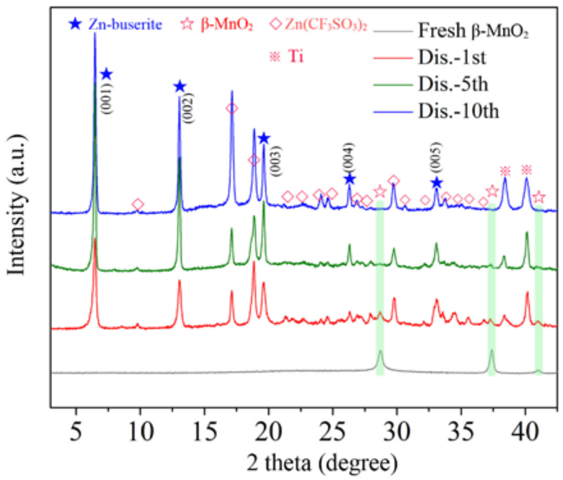

Supplementary Figure 6 | Analysis of discharged product. XRD patterns of β-MnO_2 electrodes after the 1st, 5th and 10th cycles. The characteristic peaks of initial β-MnO_2 could not be discernable after 10 cycles.

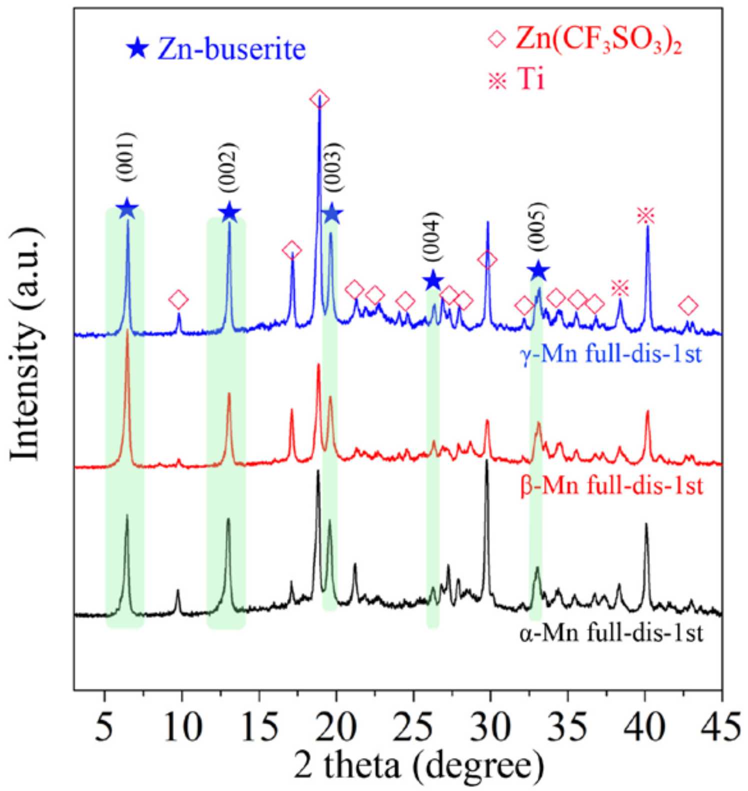

Supplementary Figure 7 | Comparsion of discharged products. XRD patterns of α-, β- and γ-MnO_2 electrodes (abbreviated as α-, β- and γ-Mn) at fully discharged state during the first cycle.

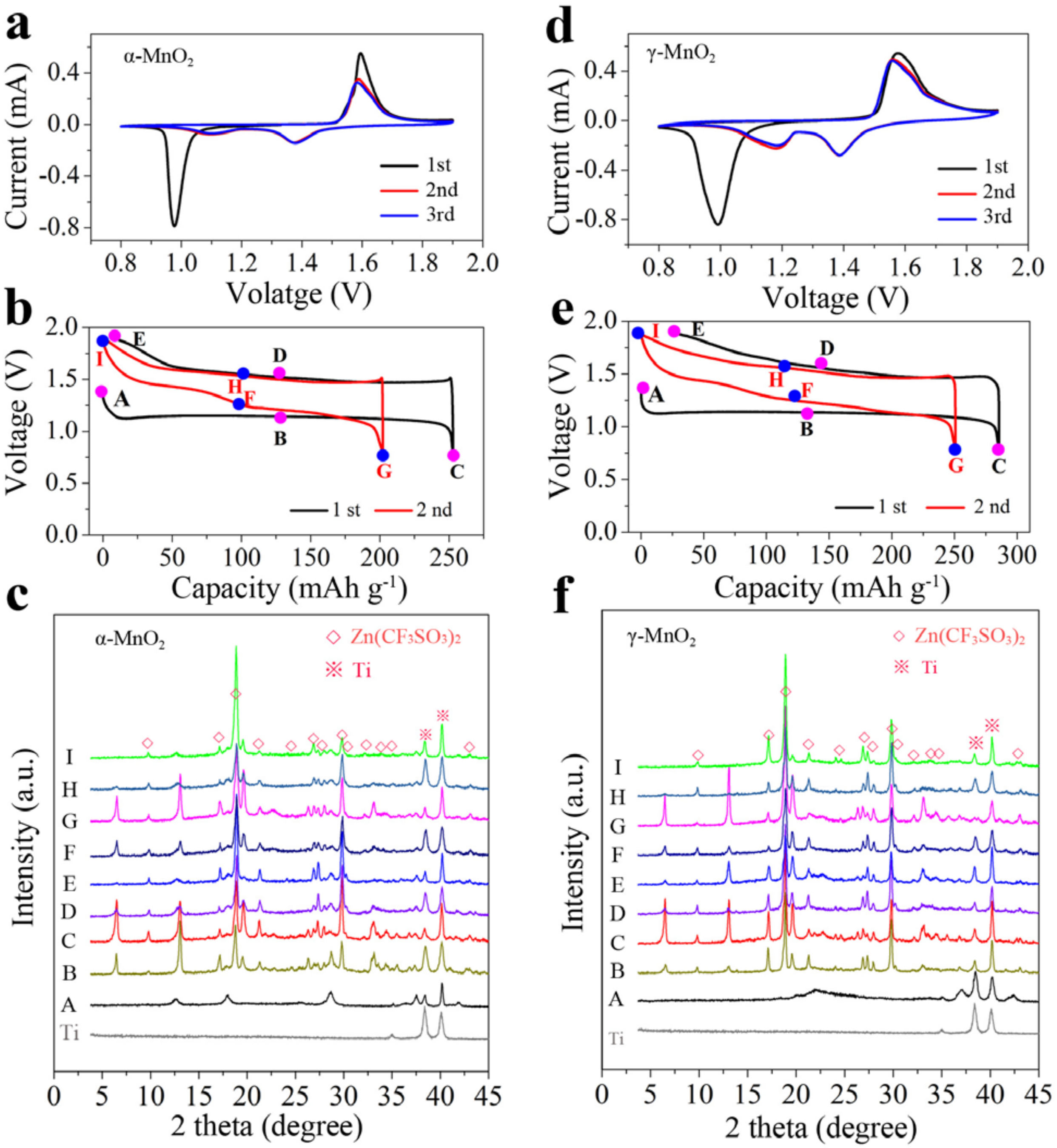

Supplementary Figure 8 | Structural evolution of α-MnO_2 and γ-MnO_2 cathodes during electrochemical reation with Zn. (**a,d**) Cyclic voltammograms, (**b,e**) typical charge/discharge curves and (**c,f**) XRD patterns of (**a-c**) α-MnO_2 and (**d-f**) γ-MnO_2 electrodes at selected states for the innitial two cycles at 0.32C. The points (A-I) marked the states respectively in Supplementary Fig. 8c,f where XRD data were collected.

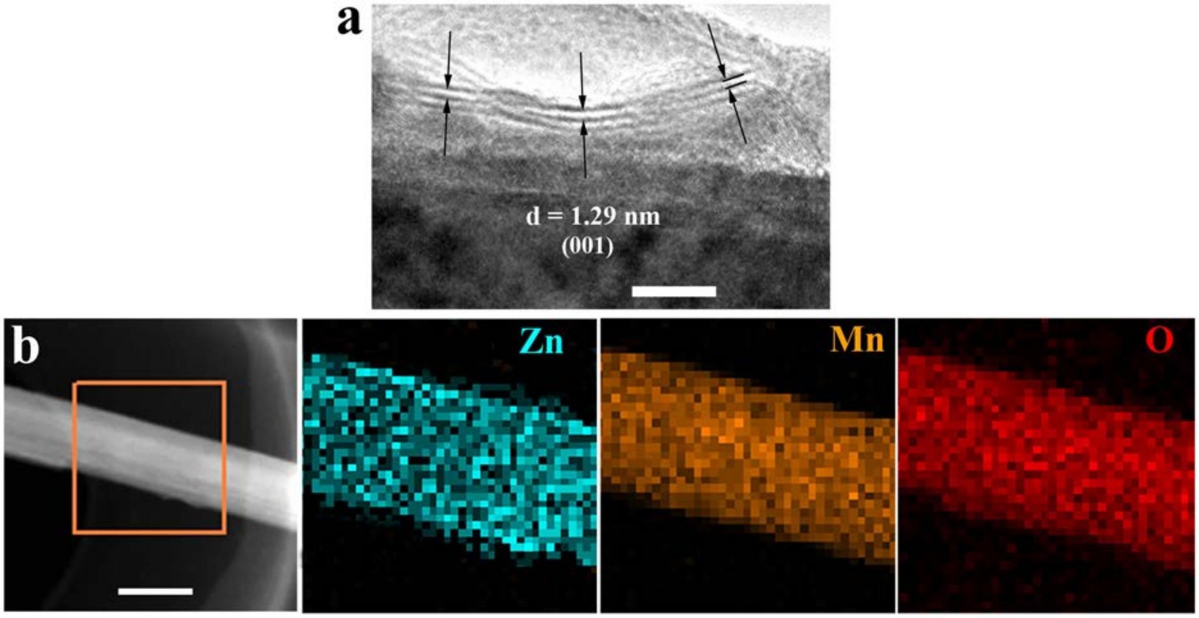

Supplementary Figure 9 | TEM and elemental analysis. (**a**) HRTEM image showing the (001) fringes and (**b**) elemental mapping in STEM of the first discharged electode. Scale bars, 10 nm (**a**) and 200 nm (**b**), respectively.

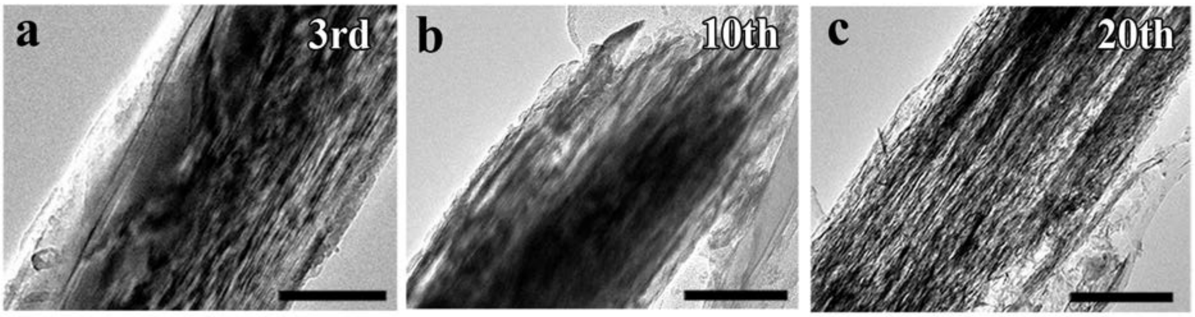

Supplementary Figure 10 | TEM characterization. TEM images of charged electrodes in 3 M $Zn(CF_3SO_3)_2$ electrolyte after (**a**) 3, (**b**) 10 and (**c**) 20 cycles. Scale bar, 100 nm.

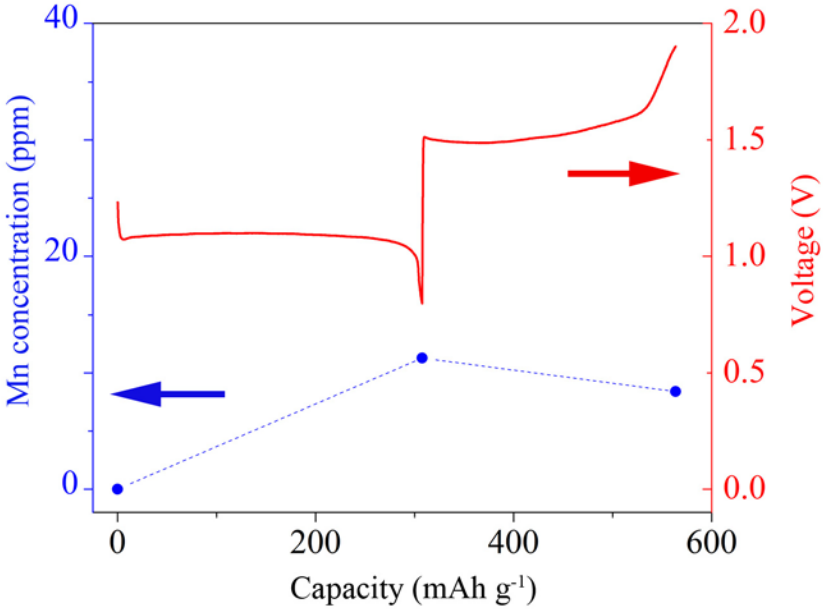

Supplementary Figure 11 | ICP analysis. Elemental analysis of dissolved Mn^{2+} ions in the aqueous electrolyte during cycling. The relative standard deviation (RSD) values are 1.7%, 2.9% and 3.1% for the intial, fully discharged, and fully charged state electrodes, respectively. Calculation of the dissolved Mn amount is described in Supplementary Note 1.

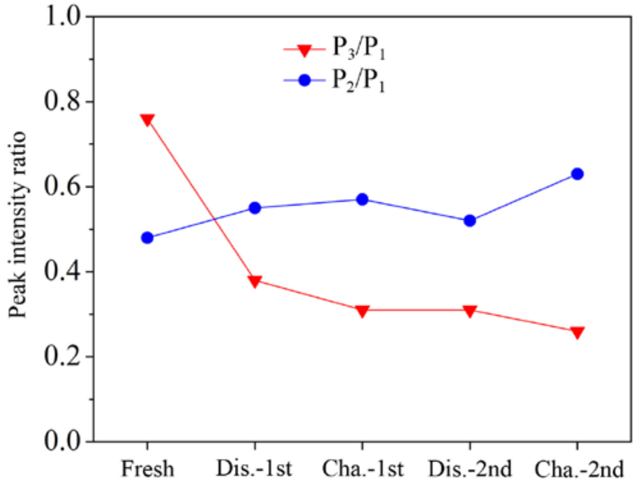

Supplementary Figure 12 | Peak intensity ratio based on the results of EXAFS spectra. P_1, P_2 and P_3 represent the Mn-O, $Mn\text{-}Mn_{edge}$ and $Mn\text{-}Mn_{corner}$ peaks (shown in Figure 4), respectively.

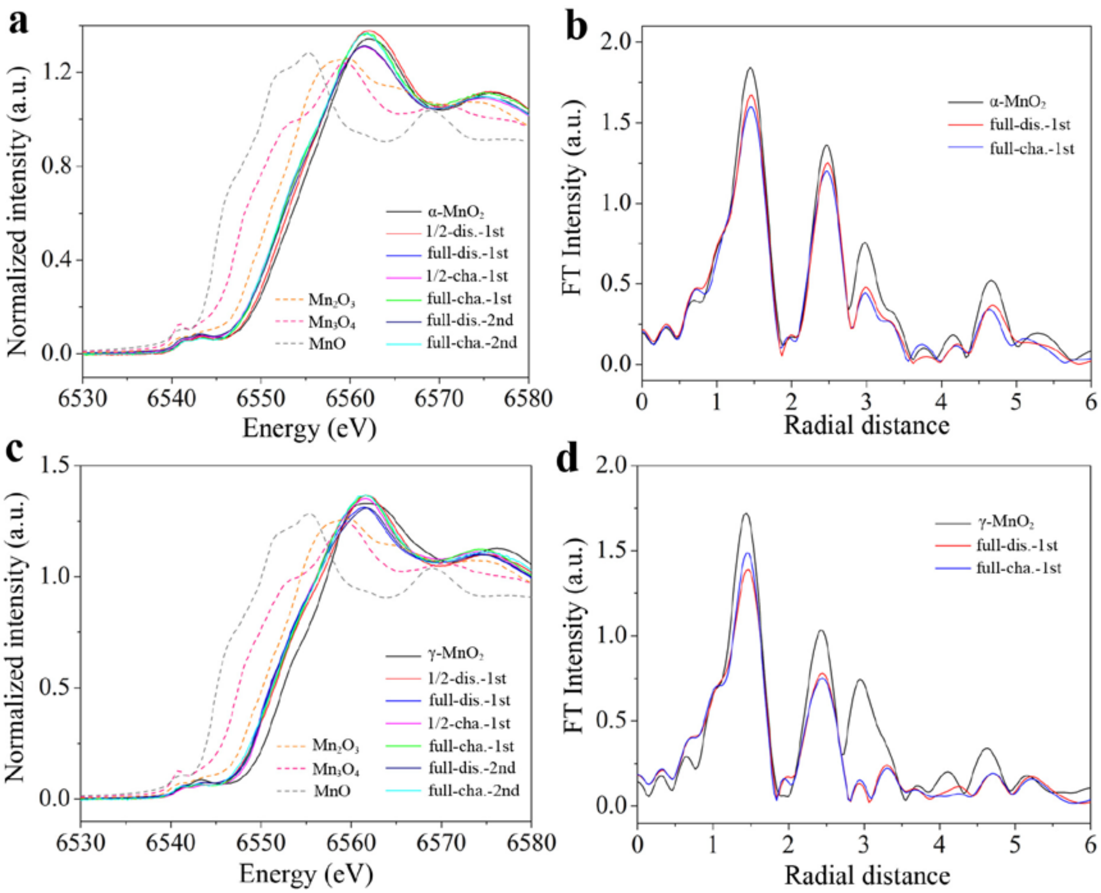

Supplementary Figure 13 | XAS characterization of α-MnO_2 and γ-MnO_2 electrodes. (**a,c**) Mn-K edge XANES patterns and (**b,d**) EXAFS spectra of (**a,b**) α-MnO_2 and (**c,d**) γ-MnO_2 electrodes at selected discharge/charge states.

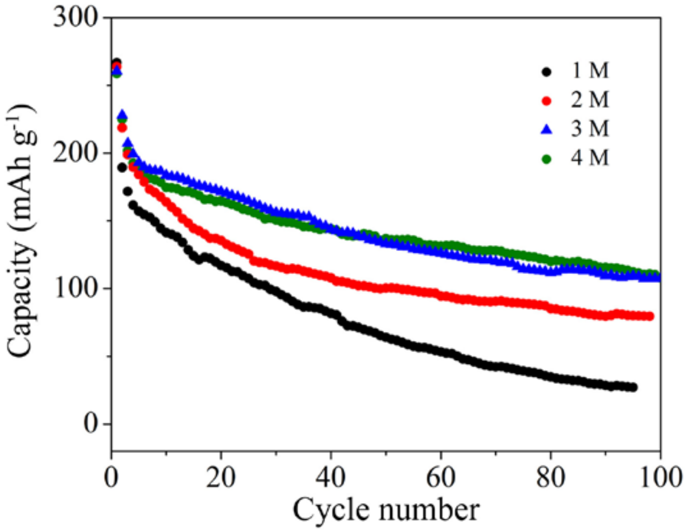

Supplementary Figure 14 | Optimization of electrolyte concentration. Cycling performance comparison of Zn-MnO_2 cells using $Zn(CF_3SO_3)_2$ electrolytes with different concentrations (1–4 M) at 0.65C.

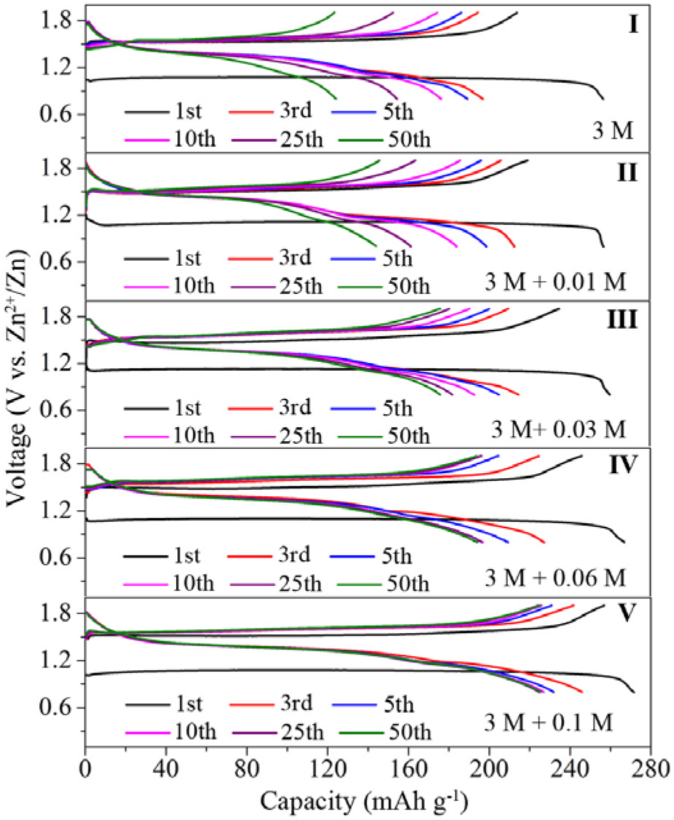

Supplementary Figure 15 | Formulation of additive concentration for Zn-MnO$_2$ batteries. Discharge/discharge curves of Zn-MnO$_2$ cells at 0.65C in (**I**) 3 M Zn(CF$_3$SO$_3$)$_2$ electrolyte and 3 M Zn(CF$_3$SO$_3$)$_2$ with (**II**) 0.01 M, (**III**) 0.03 M, (**IV**) 0.06 M and (**V**) 0.1 M Mn(CF$_3$SO$_3$)$_2$ additives (abbreviated as 3 M, 3 M + 0.01 M, 3 M + 0.03 M, 3 M + 0.06 M, and 3 M + 0.1 M, respectively).

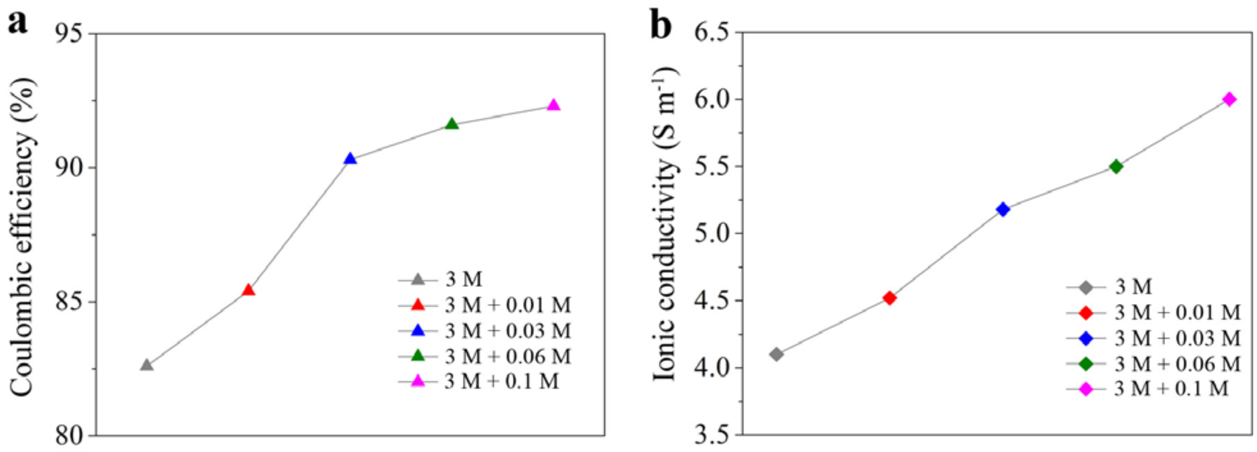

Supplementary Figure 16 | Electrolyte characterization. (**a**) The initial Coulombic efficiencies of β-MnO_2 cathode in 3 M $Zn(CF_3SO_3)_2$ electrolytes with different concentrations of $Mn(CF_3SO_3)_2$ additive. (**b**) Ionic conductivities of the corresponding electrolytes.

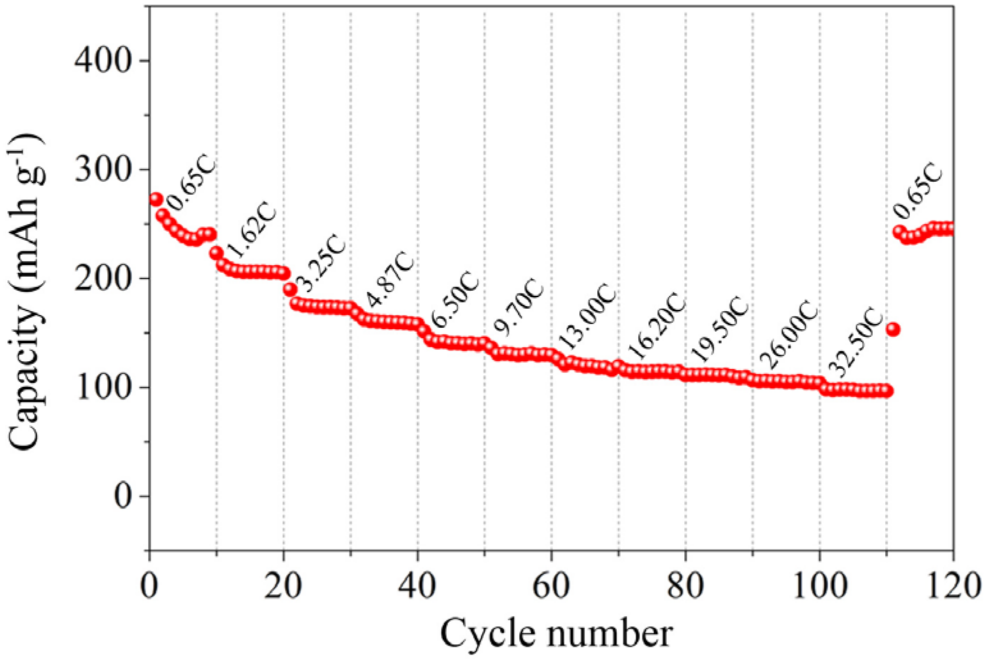

Supplementary Figure 17 | Rate performance of β-MnO_2. The current rate was gradually increased from 0.65C, 1.62C, 3.25C, 6.50C, 16.20C, 26.00C and 32.50C respectively.

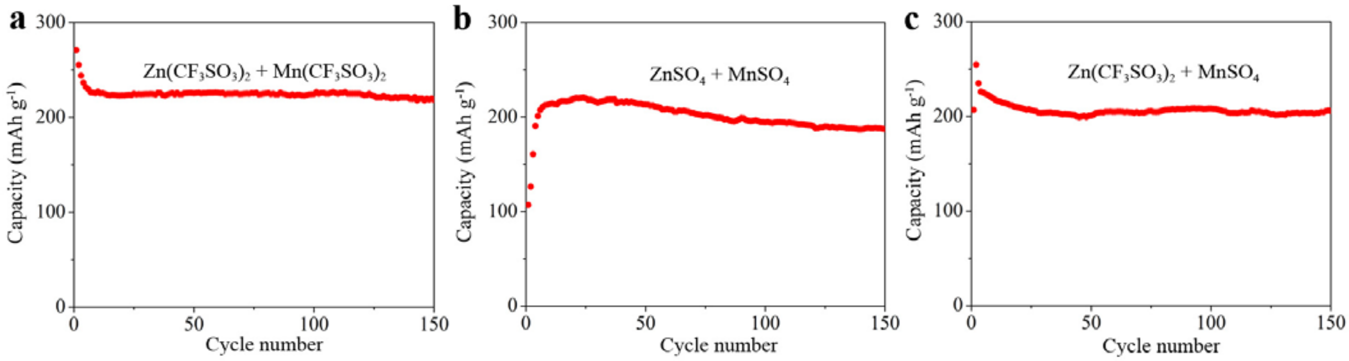

Supplementary Figure 18 | Electrochemical test. The cycling performance of Zn-MnO_2 cell using (**a**) 3 M $Zn(CF_3SO_3)_2$ + 0.1 M $Mn(CF_3SO_3)_2$, (**b**) 3 M $ZnSO_4$ + 0.1 M $MnSO_4$ electrolyte and (**c**) 3 M $Zn(CF_3SO_3)_2$ + 0.1 M $MnSO_4$ electrolytes at 0.65C. Compared with $ZnSO_4$ + $MnSO_4$ (110 mAh g^{-1}) and $Zn(CF_3SO_3)_2$ + $MnSO_4$ (205 mAh g^{-1}) counterparts, the cell in $Zn(CF_3SO_3)_2$ + $Mn(CF_3SO_3)_2$ electrolyte shows much higher initial discharge capacity of 275 mAh g^{-1} and more stable cycling performance with higher reversible capacity of 225 mAh g^{-1} after 150 cycles.

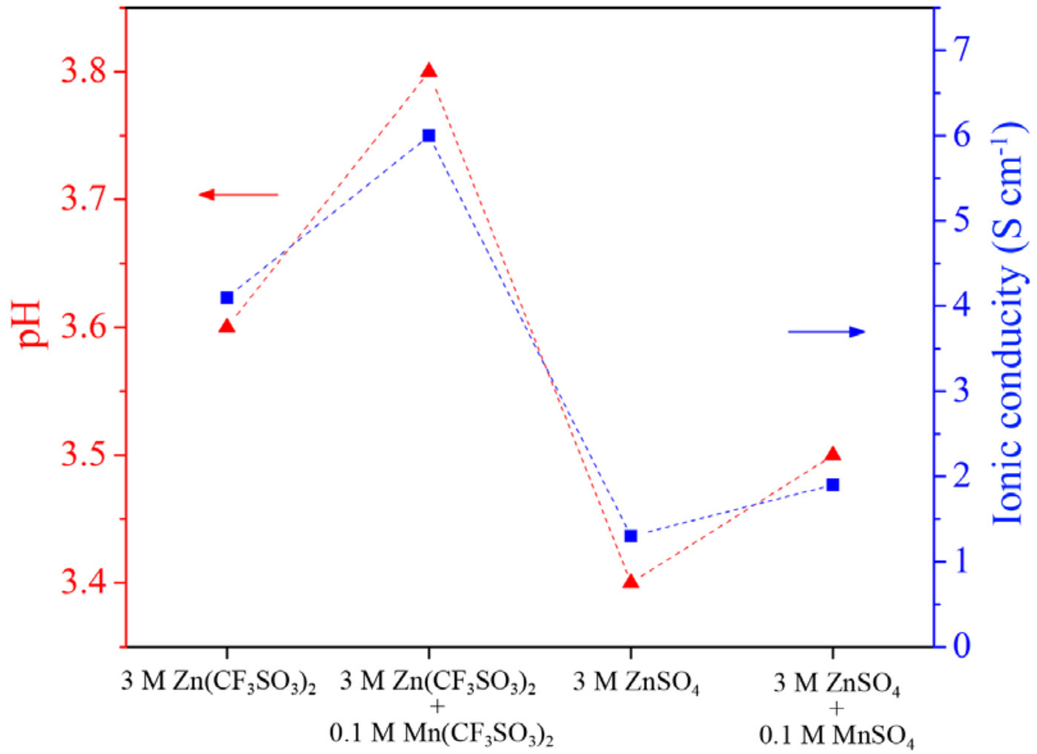

Supplementary Figure 19 | Characterization of aqueous electrolytes. pH value and ionic conductivity of 3 M $Zn(CF_3SO_3)_2$, 3 M $Zn(CF_3SO_3)_2$ + 0.1 M $Mn(CF_3SO_3)_2$, 3 M $ZnSO_4$, and 3 M $ZnSO_4$ + 0.1 M $MnSO_4$ electrolytes.

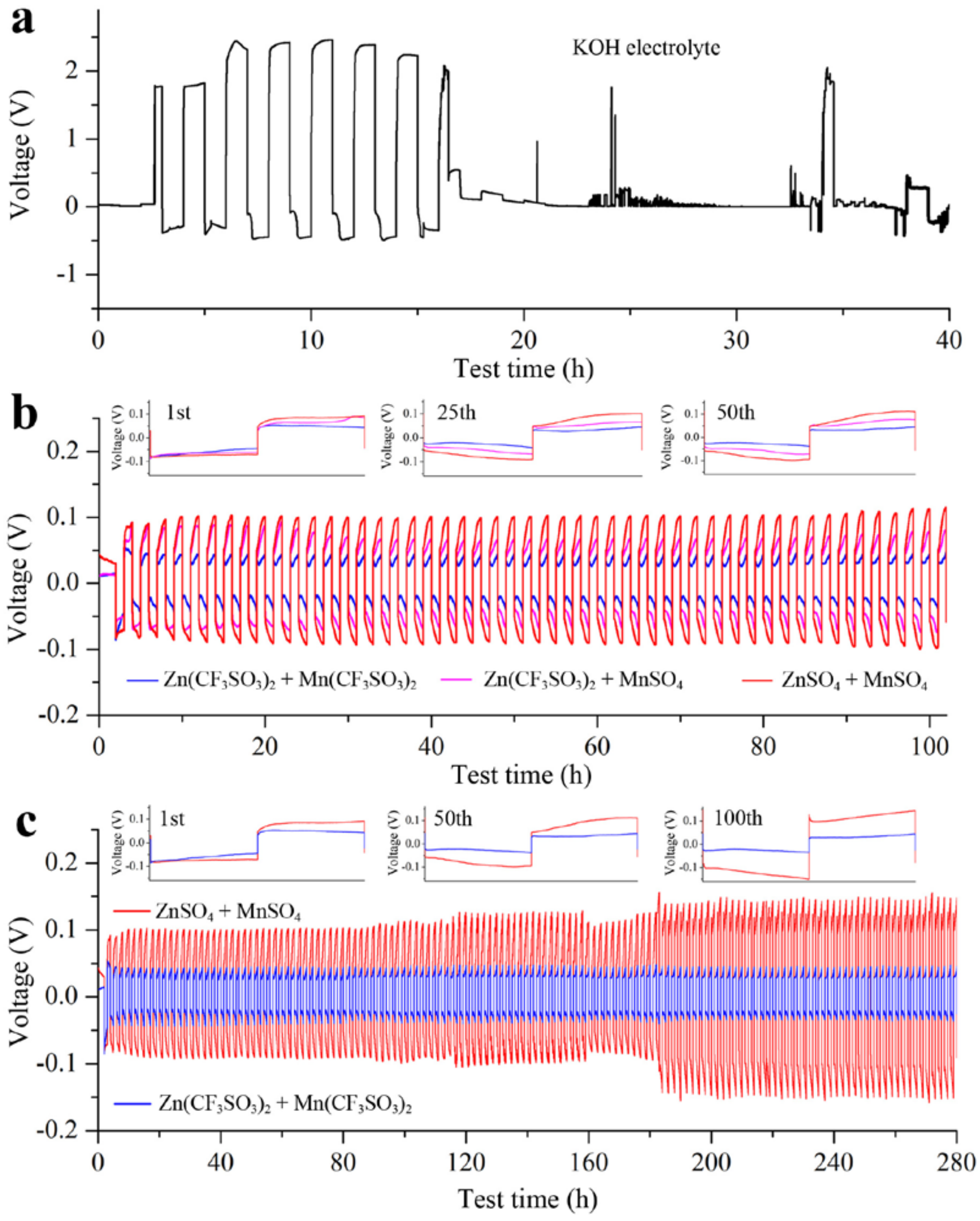

Supplementary Figure 20 | Zn plating/stripping test. Galvanostatic cycling of Zn/Zn symmetrical cells at a fixed current density of 0.1 mA cm^{-2} in (**a**) 45wt.% KOH and (**b**) 3 M $Zn(CF_3SO_3)_2$ + 0.1 M $Mn(CF_3SO_3)_2$, 3 M $Zn(CF_3SO_3)_2$ + 0.1 M $MnSO_4$ and 3 M $ZnSO_4$ + 0.1 M $MnSO_4$ electrolytes. Insets enlarge the voltage profiles of the 1st, 25th and 50th cycles. (**c**) Long-term cycling performance of Zn

plating/stripping in 3 M $Zn(CF_3SO_3)_2$ + 0.1 M $Mn(CF_3SO_3)_2$ and 3 M $ZnSO_4$ + 0.1 M $MnSO_4$ electrolytes at 0.1 mA cm^{-2}. Insets show detailed voltage profiles of the 1st, 50th and 100th cycles, respectively. Additional discussion is given in Supplementary Note 2.

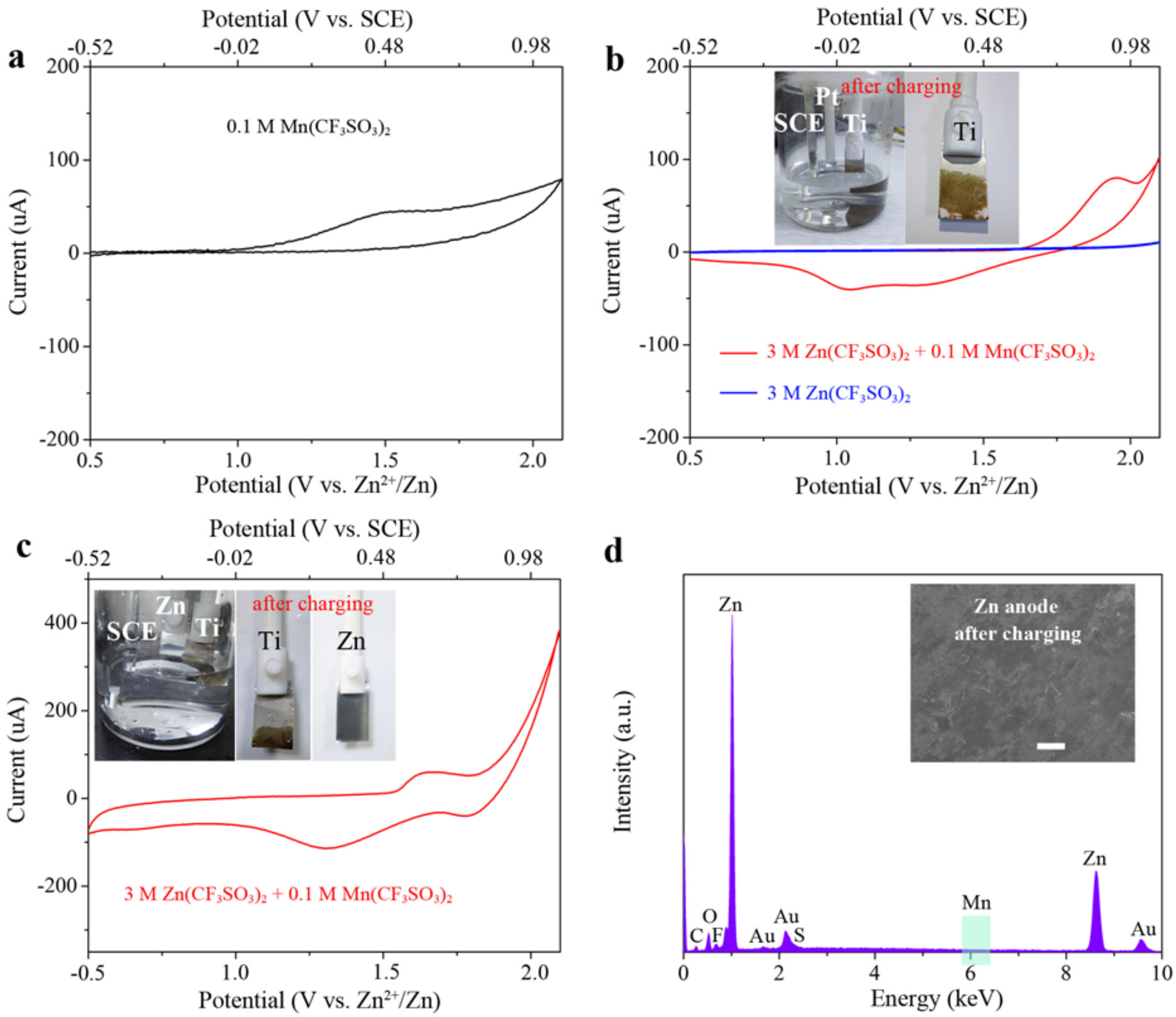

Supplementary Figure 21 | Electrochemical behaviour of Mn^{2+} additive in three-electrode cell. CV test in (**a**) 0.1 M $Mn(CF_3SO_3)_2$ solution and (**b**) 3 M $Zn(CF_3SO_3)_2$ electrolytes with/without 0.1 M $Mn(CF_3SO_3)_2$ additive, using three-electrode cell with Ti foil as the working electrode, Pt plate (**a,b**) or Zn foil (**c**) as the counter electrode, and saturated calomel electrode (SCE) as the reference electrode. Insets show the digital photo of the corresponding three-electrode cells, Zn and Ti foil electrode after charging. For convenience, the potential scale is also converted to the Zn^{2+}/Zn reference. (**d**) SEM and EDS analysis of Zn electrode after charging in 3 M $Zn(CF_3SO_3)_2$ + 0.1 M $Mn(CF_3SO_3)_2$ electrolyte. Scale bar, 2 μm. Additional discussion is given in Supplementary Note 3.

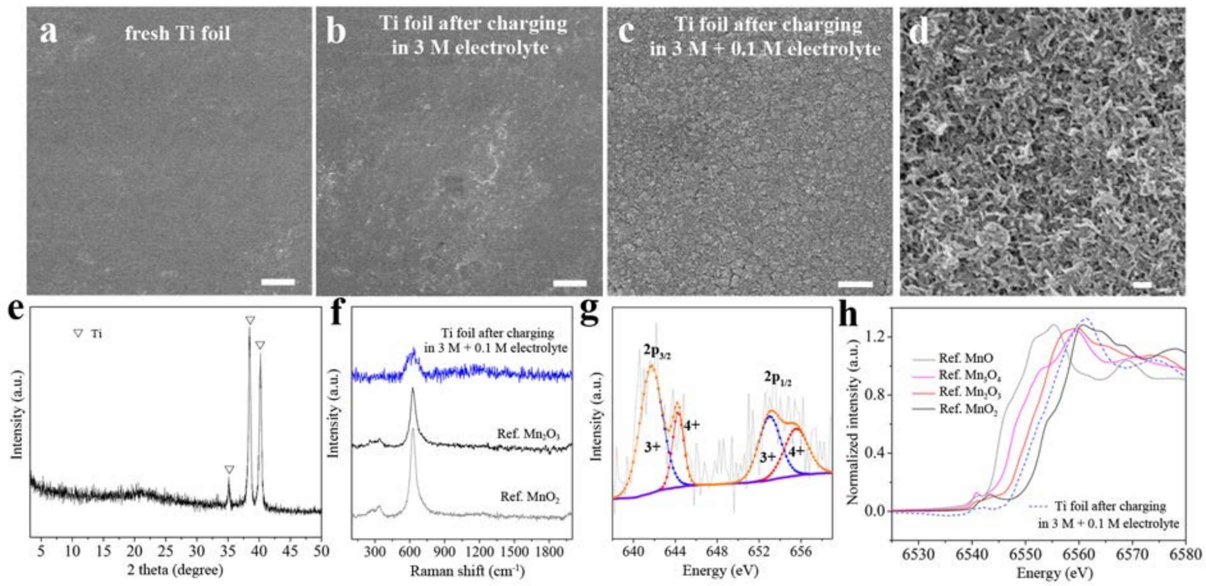

Supplementary Figure 22 | Electrolysis product of Mn^{2+} on Ti foil. SEM images of (**a**) fresh Ti working electrode and Ti electrode after charging in 3 M $Zn(CF_3SO_3)_2$ electrolyte (**b**) without or with (**c,d**) 0.1 M $Mn(CF_3SO_3)_2$ electrolyte. (**e**) XRD, (**f**) Raman, (**g**) XPS and (**h**) Mn-K edge XANES curves of Mn^{2+} electrolytic species on Ti electrode. Scale bars, 1 μm (**a–c**) and 100 nm (**d**), respectively.

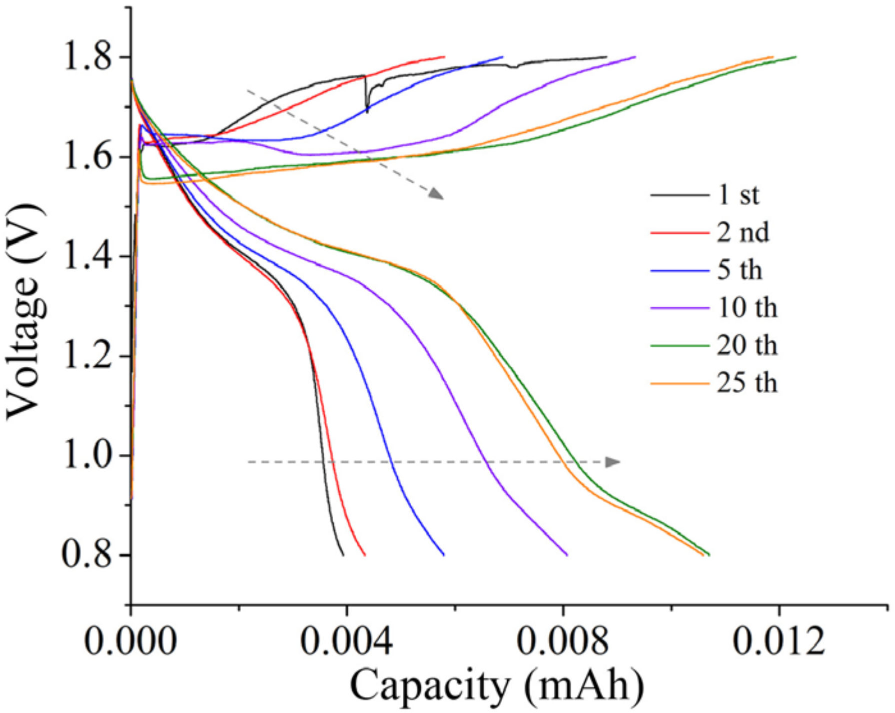

Supplementary Figure 23 | Electrochemical test. Charge/discharge profiles of Zn/Ti cells at 10 μA cm^{-2} using 3 M $Zn(CF_3SO_3)_2$ electrolyte with 0.1 M $Mn(CF_3SO_3)_2$ additive. The electrolytic MnO_x from Mn^{2+} additive could deliver a capacity of 0.011 mAh in a Zn/Ti cell, which is around 2.4% for the deliverable capacity ($2 \text{ mg} \times 225 \text{ mAh/g} \times 10^{-3} = 0.45 \text{ mAh}$) of the active material.

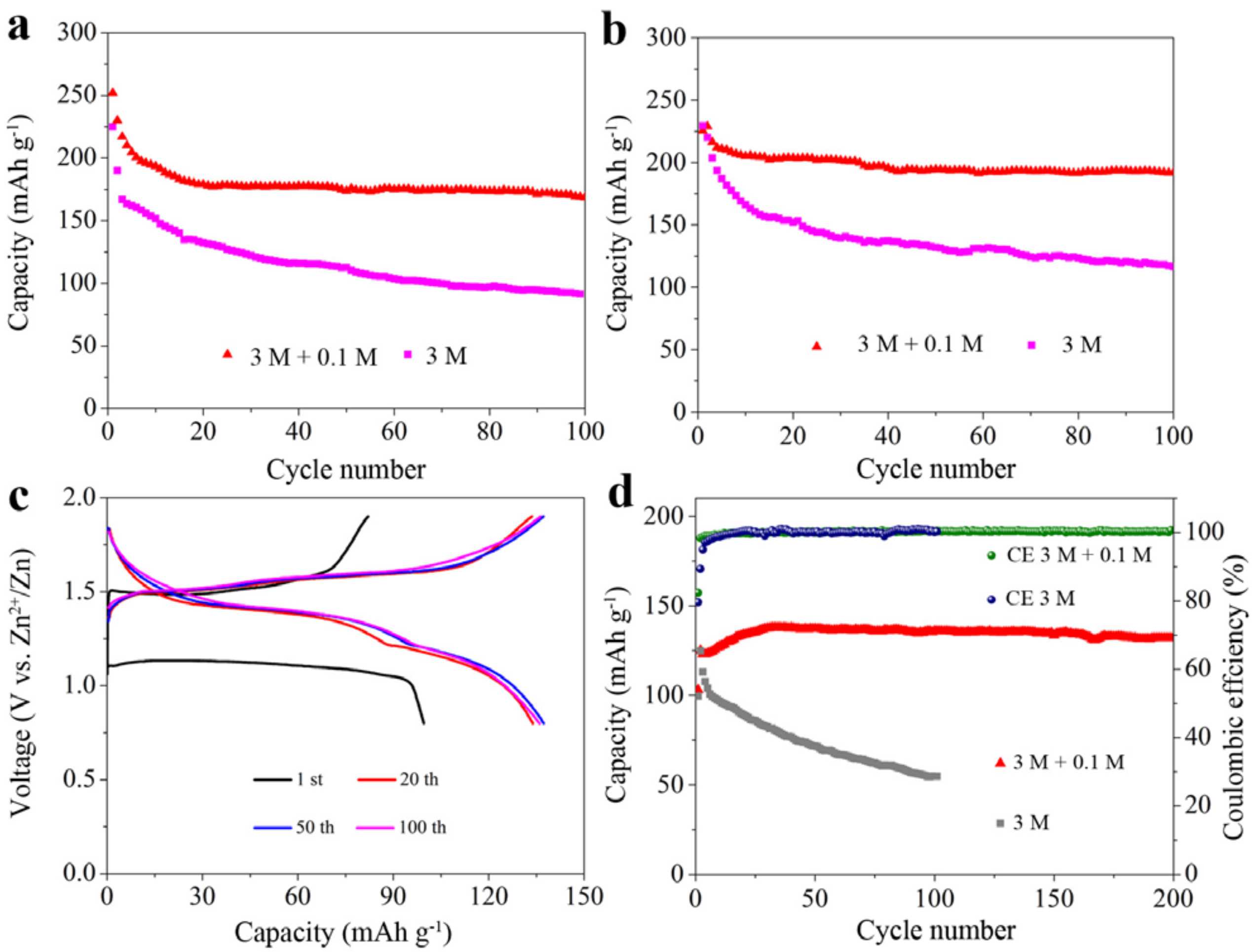

Supplementary Figure 24 | Electrochemical performance of as-prepared α-MnO_2, γ-MnO_2 and commercial β-MnO_2 electrodes. The cycling performance of (**a**) α-MnO_2 and (**b**) γ-MnO_2 cathodes using 3 M $Zn(CF_3SO_3)_2$ electrolytes with/without 0.1 M $MnCF_3SO_3)_2$ at 0.65C. (**c**) The typical charge/discharge curves in 3 M $Zn(CF_3SO_3)_2$ + 0.1 M $MnCF_3SO_3)_2$ electrolyte and (**d**) cycling performance of commercial β-MnO_2 cathode using 3 M $Zn(CF_3SO_3)_2$ electrolyte with/without 0.1 M $MnCF_3SO_3)_2$ at 0.65C. 3 M $Zn(CF_3SO_3)_2$, 3 M $Zn(CF_3SO_3)_2$ electrolytes with 0.1 M $MnCF_3SO_3)_2$, and Coulombic efficiency are abbreviated as 3 M, 3 M + 0.1 M and CE, respectively.

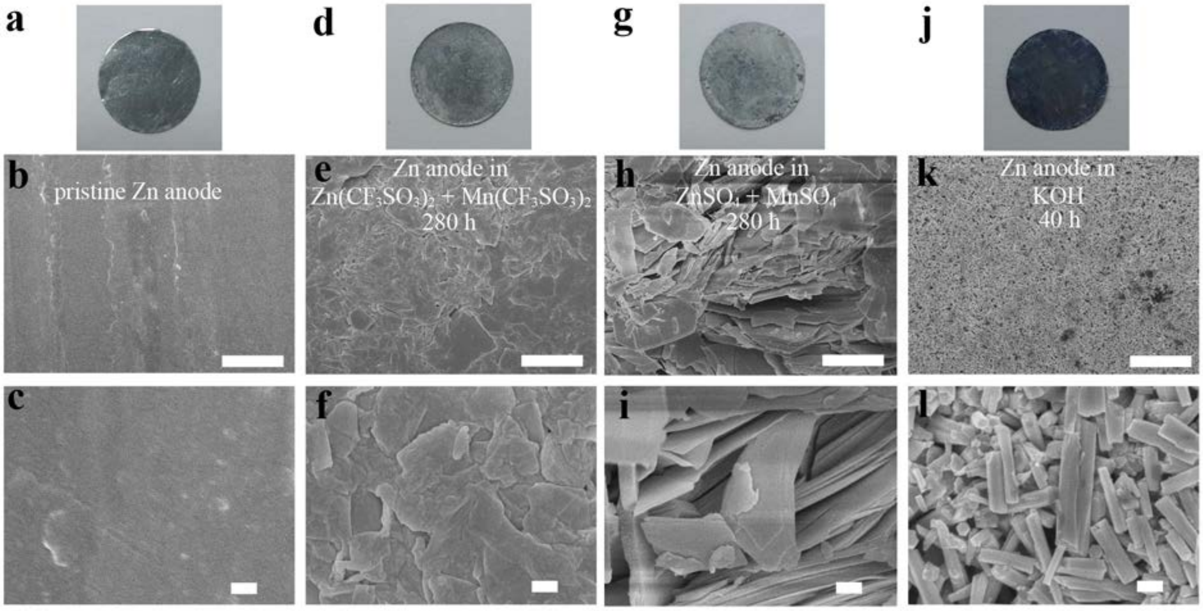

Supplementary Figure 25 | Characterization of Zn anode. The **(a,d,g,j)** optical images and (**b,c,e,f,h,i,k**,**l**) SEM images of (**a-c**) pristine Zn anode and cycled Zn anodes in (**d-f**) 3 M $Zn(CF_3SO_3)_2$ + 0.1 M $Mn(CF_3SO_3)_2$, (**g-i**) 3 M $ZnSO_4$ + 0.1 M $MnSO_4$ and (**j-l**) 45wt.% KOH electrolytes after Zn plating/stripping tests. Scale bars, 5 μm (**b,e,h,k)** and 200 nm (**c,f,i,l**), respectively.

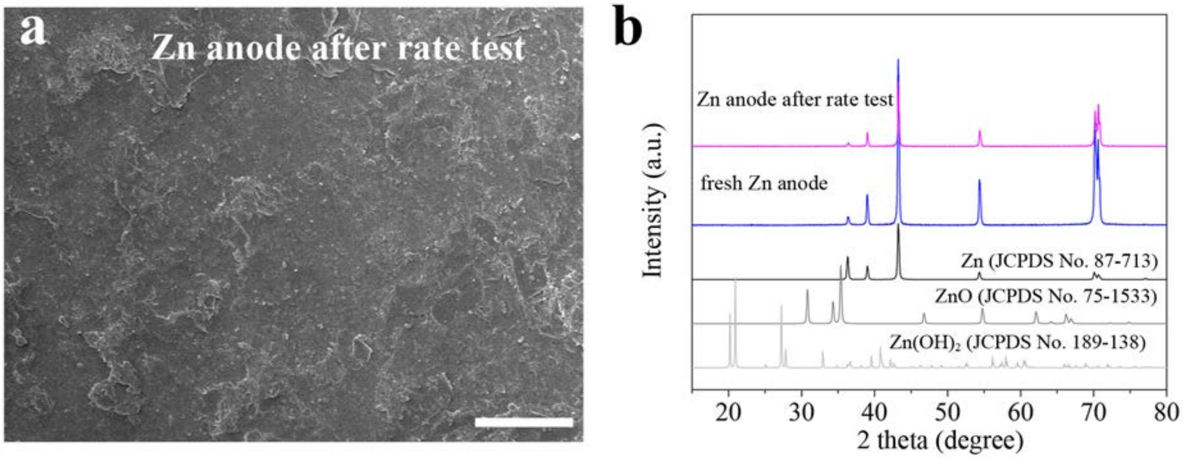

Supplementary Figure 26 | Characterization of Zn anode in Zn-MnO_2 cell. (**a**) SEM image (Scale bar, 10 μm) and (**b**) XRD pattern of cycled Zn anode in 3 M $Zn(CF_3SO_3)_2$ + 0.1 M $Mn(CF_3SO_3)_2$ electrolyte after 120 cycles of rate test (Supplementary Fig. 17).

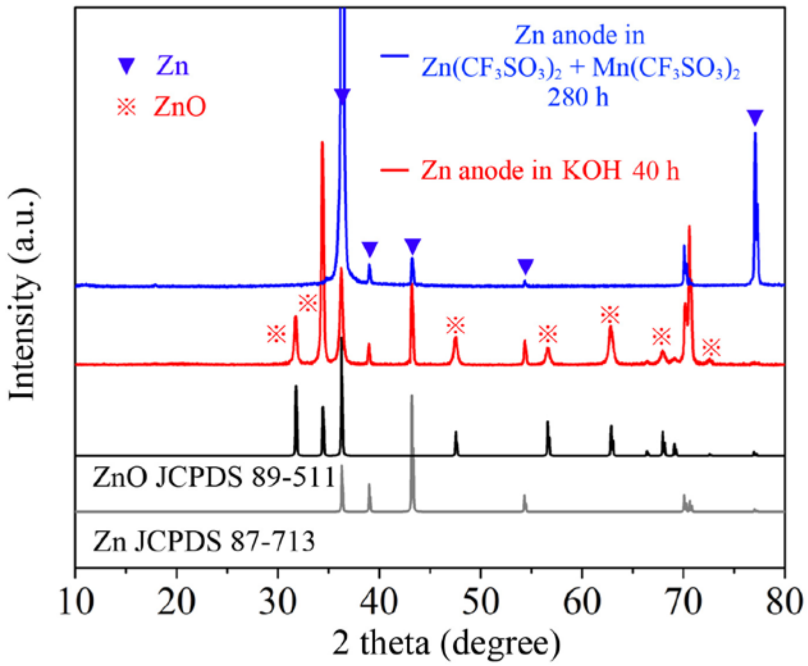

Supplementary Figure 27 | Characterization of Zn anode. The XRD patterns of cycled Zn anodes in 3 M $Zn(CF_3SO_3)_2$ + 0.1 M $Mn(CF_3SO_3)_2$ and 45wt.% KOH electrolytes after Zn plating/stripping test (Supplementary Fig. 20).

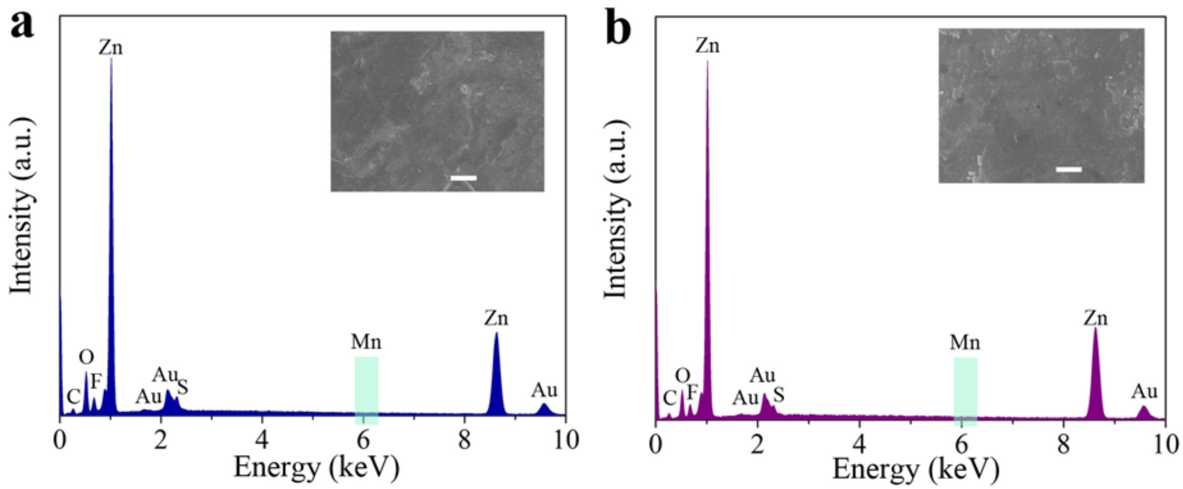

Supplementary Figure 28 | Analysis of Zn anode in Zn-MnO_2 cell. SEM-EDS analysis of Zn anode after charging in (**a**) 3 M $Zn(CF_3SO_3)_2$ and (**b**) 3 M $Zn(CF_3SO_3)_2$ + 0.1 M $Mn(CF_3SO_3)_2$ electrolytes. Insets show the corresponding SEM images. Scale bar, 1 μm. When the cell was charged to 1.9 V, negligible amount of electro-reduced Mn could be detected on the surface of Zn anode.

Supplementary Table 1. Fitted impendence parameters of re-obtained cathodes after 10 cycles in 3 M $Zn(CF_3SO_3)_2$ electrolyte with/without 0.1 M $Mn(CF_3SO_3)_2$ additive.

	In 3 M $Zn(CF_3SO_3)_2$ electrolyte with 0.1 M $Mn(CF_3SO_3)_2$ additive		In 3 M $Zn(CF_3SO_3)_2$ electrolyte	
	Value	Error (%)	Value	Error (%)
R_s	4.5 Ω	3.59	8.0	7.13
R_i	6.0 Ω	5.64	---	---
R_{ct}	25 Ω	2.51	350	3.94
Z_w	124.7 Ω	5.29	1200	9.83

Supplementary Note 1. The amount of dissolved Mn during discharge/charge was determined by ICP-AES. Take the fully discharged cell as an example. After disassembled the discharged cell, we collected the cathode, anode and electrolyte-soaked sparator, which were immersed in 10 mL deionized water for 12 h at room temperature. The obtained solution was used for ICP-AES measurement. Based on the ICP data, the concentration of dissolved Mn was 11.29 ppm. Thus, the amount of Mn of electrolyte was approximately 2.05 μmol (11.29 mg/L × 10 mL ÷ 54.94 g/mol = 2.05 μmol). A typical cell contains 2 mg β-MnO_2 (M = 87 g mol^{-1}) in the cathode, giving Mn amount of 23 μmol. Therefore, the fraction of Mn dissolved in the electrolyte out of the original Mn in β-MnO_2 is ~8.9%.

Supplementary Note 2. The Zn plating/stripping performance of aqueous electrolytes was performed in Zn/Zn symmetric cells. The symmetric cell configuration is commonly used in battery chemistries (e.g., Zn-based[1,5-7] and Li-based[8-10] systems) for investigating the intrinsic properties of electrolytes, as it permits high rates of charge/discharge and eliminating the effect of undesired reactions associated with the counter electrode. In the alkaline electrolyte, there is severe polarization of Zn stripping and plating. The $CF_3SO_3^-$-based electrolyte exhibits much smaller polarization of Zn stripping/plating and ~100% Coulombic efficiency, whereas SO_4^{2-}-based electrolyte shows apparently larger overpotential and augment of charge/discharge voltage separation. This result indicates that the $CF_3SO_3^-$-based electrolyte features fast kinetics and good reversibility of Zn plating/stripping. Furthermore, in 3 M $Zn(CF_3SO_3)_2$ + 0.1 M $Mn(CF_3SO_3)_2$ electrolyte, flat voltage plateau at discharging/charging states can be retained up to 280 h without obvious increase in hysteresis, indicating extremely high stability.

Supplementary Note 3. Notably, the standard electrode potential for the electro-reduction of Mn^{2+} in aqueous solution is E^{θ} = -1.18 V vs. SHE (-0.40 V vs. Zn^{2+}/Zn) based on the equation $Mn^{2+} + 2e^- = Mn$.

The required theoretical potentials to form Mn in 0.1 M Mn^{2+}-containing solution is calculated to be -0.43 V. It is unlikely that Mn is deposited on the zinc anode between 0.8–1.9 V, which is the working voltage window for the Zn-MnO_2 cell. The CV results indicate that in 0.1 M $Mn(CF_3SO_3)_2$ solution (pH 6.0), the onset anodic peak is at around 1.20 V, which is assigned to the electrolytic oxidation of Mn^{2+} (Supplementary Fig. 21a). In 3 M $Zn(CF_3SO_3)_2$ electrolyte with 0.1 M additive (pH 3.8), the onset potential of Mn^{2+} oxidation shifts to a higher potential at 1.70 V. After charging, the brown deposit was obviously observed on the surface of Ti (inset), which is further analyzed in Supplementary Fig. 22. Meantime, during cathodic sweep, the broad peak located at 1.35 V could be assigned to the insertion of Zn^{2+} ions into the generated MnO_x. As expected, no peaks are observed in 3 M $Zn(CF_3SO_3)_2$, indicating the high chemical stability within the working voltage window (0.8–1.9 V) of Zn-MnO_2 cell. Negligible Mn could be detected from EDS analysis of the Zn anode, consistent with the expectation from the estimated potential of Mn reduction.

Supplementary Methods:

Synthesis. All chemicals were of analytical grade without further purification. $KMnO_4$, $MnSO_4$•H_2O and $(NH_4)_2S_2O_8$ were all purchased from *Alfa Aesar*. Deionized water was used throughout. In a typical synthesis of α-MnO_2, 11 mM $KMnO_4$ and 15 mM $MnSO_4$ were mixed in 80 mL water with continuous stirring to form a homogeneous mixture. The obtained mixture was then transferred into a Teflon-lined stainless steel autoclave and heated at 140 ^oC for 12 h. To synthesize γ-MnO_2, 10 mM $MnSO_4$ and 10 mM $(NH_4)_2S_2O_8$ were mixed with 60 mL water; the mixture was hydrothermally treated at 90 ^oC for 24 h. The obtained samples were collected, washed and dried with procedures similar to that of β-MnO_2.

Soft-packed batteries preparation. The cathode was fabricated by mixing MnO_2 (85wt%), Super P carbon (10wt%) and polyvinylidene fluoride (PVDF, 5wt%) in N-methyl-2-pyrrolidone (NMP) solvent. The slurry was spread onto both sides of stainless steel foil by a coating machine and then dried in a vacuum oven at 80 ^oC for 12 h. The electrode was pressed under 15 MPa and was weighed. The loading mass is ~12 mg cm^{-2} on one side of the current collector. For the anode, zinc powder (90%), carbon black (5%) and PVDF (5%) were mixed in NMP to obtain the slurry, which was then coated on the stainless steel foil and dried in a vacuum oven at 80 ^oC for 12 h. The balancing mass ratio of cathode and anode materials was set as 2.5:1 based on the theoretical specific capacity of cathode (308 mAh g^{-1}) and anode (824 mAh g^{-1}). The electrodes were cut into 9.5 cm × 8 cm rectangular shape for soft-packed cells. The electrolyte is 3M $Zn(CF_3SO_3)_2$ with 100 mM $Mn(CF_3SO_3)_2$ aqueous electrolyte, and filter paper is the separator. The soft-packed battery was directly assembled in ambient air and the whole mass was 27.8 g. The pouch-type battery adopts internal configuration with 6 anode–separator–cathode stacks. The battery was galvanostatically tested using a LAND-CT2001A battery-testing instrument. The voltage range was 0.8–1.9 V and the current was 0.72 A.

Supplementary References

1. Pan, H. *et al.* Reversible aqueous zinc/manganese oxide energy storage from conversion reactions. *Nat. Energy* **1**, 16039 (2016).
2. Lee, B. *et al.* Electrochemically-induced reversible transition from the tunneled to layered polymorphs of manganese dioxide. *Sci. Rep.* **4**, 6066 (2014).
3. Xu, C., Li, B., Du, H. & Kang, F. Energetic zinc ion chemistry: The rechargeable zinc ion battery. *Angew. Chem. Int. Ed.* **51**, 933-935 (2012).
4. Alfaruqi, M. H. *et al.* Electrochemically induced structural transformation in a γ-MnO_2 cathode of a high capacity zinc-ion battery system. *Chem. Mater.* **27**, 3609-3620 (2015).
5. Liu, Z., Pulletikurthi, G., & Endres, F. A Prussian Blue/Zinc secondary battery with a bio-ionic liquid−water mixture as electrolyte. *ACS Appl. Mater. Interf.* **8**, 12158−12164 (2016).
6. Parker, J. F. *et al.* Wiring zinc in three dimensions re-writes battery performance–dendrite-free cycling. *Energy Environ. Sci.* **7**, 1117–1124 (2014).
7. Simons, T. J., MacFarlane, D. R., Forsyth, M. & Howlett, P. C. Zn electrochemistry in 1-Ethyl-3-Methylimida zolium and N-Butyl- N-Methylpyrrolidinium dicyanamides: Promising new rechargeable Zn battery electrolytes. *ChemElectroChem* **1**, 1688–1697 (2014).
8. Burns, J. C. *et al.* Introducing symmetric Li-Ion cells as a tool to study cell degradation mechanisms. *J. Electrochem. Soc.* **158**, A1417-A1422 (2011).
9. Lu, Y., Tu, Z. & Archer, L. A. Stable lithium electrodeposition in liquid and nanoporous solid electrolytes. *Nat. Mater.* **13**, 961-969 (2014).
10. Lin, D. C. *et al.* Layered reduced graphene oxide with nanoscale interlayer gaps as a stable host for lithium metal anodes. *Nat. Nanotech.* **11**, 626-633 (2016).
